# Design of novel granulopoietic proteins by topological rescaffolding

**DOI:** 10.1371/journal.pbio.3000919

**Published:** 2020-12-22

**Authors:** Birte Hernandez Alvarez, Julia Skokowa, Murray Coles, Perihan Mir, Masoud Nasri, Kateryna Maksymenko, Laura Weidmann, Katherine W. Rogers, Karl Welte, Andrei N. Lupas, Patrick Müller, Mohammad ElGamacy

**Affiliations:** 1 Max Planck Institute for Developmental Biology, Tübingen, Germany; 2 University Hospital Tübingen, Division of Translational Oncology, Department of Hematology, Oncology, Clinical Immunology and Rheumatology, University Hospital Tübingen, Germany; 3 Friedrich Miescher Laboratory of the Max Planck Society Tübingen, Germany; 4 Heliopolis Biotechnology Ltd., London, United Kingdom; Stanford University, UNITED STATES

## Abstract

Computational protein design is rapidly becoming more powerful, and improving the accuracy of computational methods would greatly streamline protein engineering by eliminating the need for empirical optimization in the laboratory. In this work, we set out to design novel granulopoietic agents using a rescaffolding strategy with the goal of achieving simpler and more stable proteins. All of the 4 experimentally tested designs were folded, monomeric, and stable, while the 2 determined structures agreed with the design models within less than 2.5 Å. Despite the lack of significant topological or sequence similarity to their natural granulopoietic counterpart, 2 designs bound to the granulocyte colony-stimulating factor (G-CSF) receptor and exhibited potent, but delayed, in vitro proliferative activity in a G-CSF-dependent cell line. Interestingly, the designs also induced proliferation and differentiation of primary human hematopoietic stem cells into mature granulocytes, highlighting the utility of our approach to develop highly active therapeutic leads purely based on computational design.

## Abstract 2

De novo designed cytokines that activate the G-CSF receptor show that the receptor-binding information can be encoded onto stable, miniaturised protein scaffolds that possess potent granulopoietic activity; such novel proteins provide for ideal candidates for protein-based therapeutics.

## Introduction

Just as a protein fold can map to different regions of sequence space, a protein function can map to several regions of structure space [1]. A molecular function can thus be potentially achieved through a large number of possible structures, each with a range of possible sequences. While navigating these vast spaces may appear intractable, recent advances in protein design methods have enabled an unprecedented level of control of both sequence and structure [2]. This has paved the way for the rational design of functional proteins to support various applications in synthetic biology [3]. Despite their small number, studies reporting the computational design of proteins with therapeutic potential have demonstrated significant success, with roles as steroid quenchers [4], viral protein interaction inhibitors [5], influenza hemagglutinin blockers [6–8], neurotoxin blockers [8], and specific interleukin mimics [9]. All of these studies, however, have entailed one or more stages of experimental screening of mutant libraries in order to enhance the activity or to improve the designs thermostability. Other studies have reported successful direct single-step design to yield highly active proteins. Two of these studies reported the successful presentation of immunogenic epitopes from HIV [10,11]; however, these epitopes were composed of single-stretch segments, and epitope grafting to a new host protein was achieved by a straightforward backbone geometry match. Another study has shown that grafting 3 residues from 1 receptor binding site of erythropoietin (EPO) onto an alternative scaffold could generate a novel nanomolar EPO receptor inhibitor, which had micromolar IC_50_ in competitive binding assays [12]. The epitope matching strategy relied on successive residue pair distance matching and was used to graft a small 3-residue-epitope [12].

Here, we adopt an approach based exclusively on *in silico* design to conceive novel receptor activators, grafting the receptor-binding epitope of the ligand onto scaffolds with simpler topologies in single- or 2-copy configurations. Our strategy starts by disembodying discontinuous residue stretches from the cognate natural complex, using a combination of dihedral and distance matches. By preserving the 3D positions and orientations of these disembodied residues, we seek alternative scaffolds that can be adapted and rigidified to host this rudimentary pharmacophore. This not only offers a chance for radically enhanced pharmacokinetic features but can also enable the introduction of bespoke pharmacodynamic properties. As a proof of principle, we created designs with granulocyte colony-stimulating factor (G-CSF) activity. G-CSF is a cytokine that stimulates the proliferation and myeloid differentiation of hematopoietic stem and progenitor cells (HSPCs) in the bone marrow and their release into the blood stream. Recombinant human G-CSF (rhG-CSF) has demonstrated great immunotherapeutic utility due to its potent activity in the stimulation of granulopoiesis, enhancing the immunity in neutropenic patients who suffer from a severely diminished number of neutrophils due to genetic dispositions or chemotherapy [13–18]. Like most other therapeutic proteins, rhG-CSF has been clinically deployed in its native form or with a few modifications. This almost-direct adoption of a natural protein in a therapeutic context often faces major pharmaceutical development challenges, as evidenced by recombinant production yield, solubility, stability, short serum half-life, and reconstituted activity shelf-life. Engineering attempts have thus been pursued to improve rhG-CSF, spanning point-mutagenesis [19–22], PEGylation [23,24], and circularisation [25,26].

To develop novel proteins with G-CSF activity, we rescaffolded the binding epitope on G-CSF’s surface onto different host structures with smaller and simpler topologies. To evaluate the designed molecules, we analyzed their biophysical properties and determined their structures, demonstrating atomic-level agreement with the designed coordinates. Furthermore, we evaluated their potential to induce proliferation of G-CSF-dependent murine cells and proliferation and differentiation of primary human hematopoietic stem cells in vitro. Strikingly, the tested designs had binding affinities and in vitro activities corresponding to *K_d_* and *EC_50_* values ranging from nano- to micromolar as evaluated by surface plasmon resonance and cell-based biological assays, respectively. Altogether, our results demonstrate that high-precision protein design based on topological rescaffolding can offer a direct and expedited means for discovering novel potential therapeutic leads.

## Results

### Computational design

**Fig 1:**
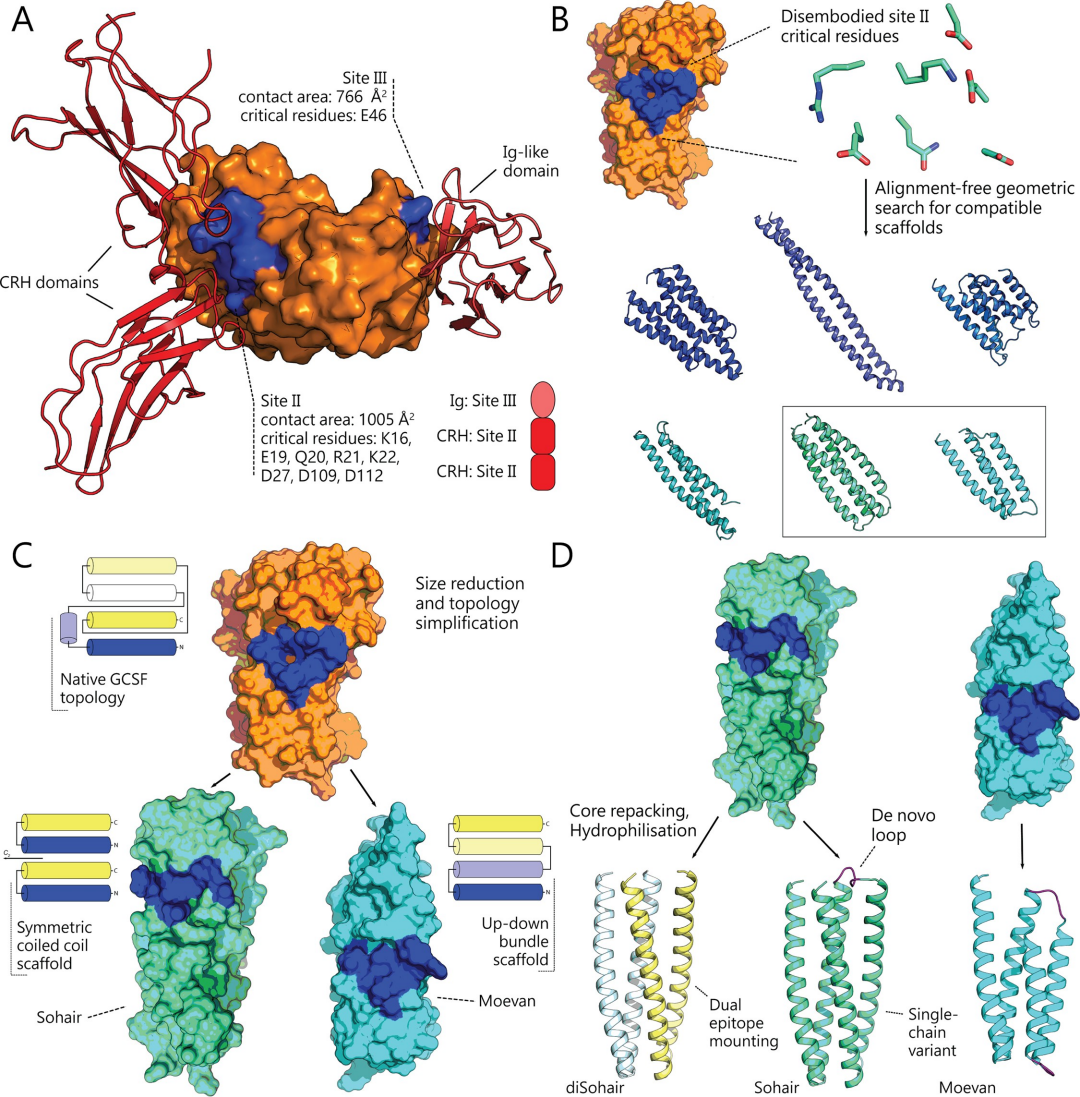
Design strategy of novel granulopoietic proteins. **(A)** Structure of the human G-CSF:G-CSFR complex (PDB: 2D9Q), showing site II and site III as the contact points for proposed receptor dimerization [40]. Surface residues at site II or site III that were reported to have a more than 2-fold impact on G-CSF’s activity are shown in blue [27]. G-CSF is shown in orange, the G-CSF receptor CRH domains in red, and the Ig-like domain in pink. **(B)** The critical residues at site II were disembodied and used as a geometric search query against the entire PDB to retrieve structurally compatible scaffolds. The top 6 compatible scaffolds structures are shown in cartoon representation. **(C)** After retrofitting the binding epitope, the most structurally stable scaffolds, named Moevan and Sohair, were identified by molecular dynamics simulations. The scaffold of Moevan and Disohair were adopted from an uncharacterized protein from *Bacillus halodurans* (PDB: 2QUP) [30] and a *de novo* designed, homodimeric, 4-helical, coiled-coil bundle (PDB: 5J73) [31], respectively. The blue-to-yellow 2D diagrams represent the topological chain paths of the G-CSF fold compared to the designs. **(D)** The retrofitted scaffolds were further optimized for their core packing and solvent-exposed residues. diSohair1 and diSohair2, dimeric variants of Sohair, were constructed to harbor 2 copies of the binding epitope in a *C_2_*-symmetric fashion, while the single-chain variant Sohair was made through *de novo* design of an additional loop across the dimeric interface of the 2 chains. G-CSF, granulocyte colony-stimulating factor; Ig, immunoglobulin; PDB, Protein Data Bank.

The sequence determinants of human G-CSF receptor binding and activation are well established. G-CSF residues at the interface region designated site II (K16, E19, Q20, R22, K23, D27, D109, and D112) are the predominant determinants of receptor binding and activation [27,28], whereas the contribution of site III has a smaller surface area and contains only a single critical binding residue (E46) (Fig 1A). This motivated us to focus on the site II binding features as a starting point for our design strategy. The 3D arrangement of the backbone atoms of G-CSF binding epitope residues in space was disembodied from the native G-CSF structure and used to screen the Protein Data Bank (PDB) for compatible hosting structures, disregarding any sequence similarity to native G-CSF (Fig 1B). The aim was to match backbone dihedrals and 3D backbone positions of the query residues to similar substructures in the PDB. To simplify the search space, the residues were assumed to lie on 2 discontinuous segments in the subject structures (i.e., segment 1: residues16 to 27, segment 2: residues 109 to 112). This allowed us to extend a previous loop-grafting routine, originally developed to find loops across discontinuous secondary structures [29], to generically search for pairs of structural segments disconnected by any number of intervening residues (see Materials and methods and S1 Text). Six hits from the PDB and their alignments were inspected more carefully: 3NKZ:A, 4A55:B, 4IUL:A, 5AQF:B, 5J73:A, and 2QUP:A. The 2 top structural hits picked from these 6 candidates were an uncharacterized protein from *Bacillus halodurans* (PDB: 2QUP) [30], and the 2L4HC2_9 design, a *de novo* designed, homodimeric, 2-helical, coiled-coil bundle (PDB: 5J73) [31]. These scaffolds had local backbone root-mean-square deviation (RMSD) values of 0.5 and 1.3 Å to the G-CSF binding epitope, respectively, and thus were chosen as design targets. Importantly, both proteins have a smaller size and a simpler topology compared to native G-CSF. In contrast to the 19 kDa G-CSF, which has the form of an up-up-down-down 4 helix bundle with 2 bundle-interrupting long loops, the 2L4HC2_9 design is a *C_2_*-symmetric dimer of a 9 kDa helical hairpin, and the protein from *B*. *halodurans* is a 13 kDa up-down 4-helix-bundle (Fig 1C). These 2 scaffolds with the G-CSF receptor binding sites modeled in were selected for further optimization in a series of design rounds aiming at hydrophobic core-repacking and hydrophilisation of solvent-exposed residues. Approximately, 10,000 hits were generated using a Rosetta routine (see Materials and methods and S1 Text). The Rosetta design routine already internally filtered hits based on the total energy score within the first Monte-Carlo looper, and the packing ruggedness score was used to filter all of the resulting decoys down to approximately 2,000 decoys (see Materials and methods and S1 Text). These decoys were further reduced to several hundred based on their CHARMM36 potential energies and finally ranked by their structural homogeneity based on molecular dynamics (MD) simulations (see Materials and methods). The most conformationally stable designs belonging to each of the 2 initial templates were selected and named Moevan and Sohair, where the latter was a single chain variant that expressed a single copy of the epitope (i.e., with no *C_2_*–symmetry).

Two topological variants were generated from the 2L4HC2_9 coiled-coil: Sohair and diSohair. Sohair is a monomeric variant with 2 coiled coils connected through a linker and displaying the binding epitope on only 1 facet of the coiled-coil bundle (Fig 1D). We sought to construct the single-chain variant Sohair with only 1 binding epitope, as MD simulations had shown that doubly displaying the binding epitope was destabilizing compared to the original template sequence. As the distance across the termini averaged around 11 Å, we sought to construct a 3-residue-loop *de novo* by testing the conformational stability by MD of every possible loop sequence built from an alphabet of 9 amino acids suitable for loop design. We simulated 9^3^ = 729 loop compositions and picked the most conformationally stable for experimental evaluation to construct a loop across the dimerisation interface in Sohair. We also wanted to create a dimeric variant, hypothesizing that a *C_2_*–symmetric site II should dimerise the receptor more effectively. Due to the destabilizing effect of carving 2 binding sites that we detected in our MD simulations, we expected that the dimeric version would be significantly less stable than the single chain variant. Therefore, we created 2 variants, attempting the stabilizing residue M24 and the disembodied Q24, in diSohair1 and diSohair2, respectively. Moevan, the other design based on structure 2QUP was only deployed in its original template topology (Fig 1D). We thus tested a total number of 4 designs experimentally: Moevan, Sohair (the single-chain, single-epitope variant), and 2 dimeric Sohair variants (diSohair1 and diSohair2). Based on the experimental results obtained from these designs, we further made a fifth construct, Moevan_t2, which is composed of 2 tandemly repeated Moevan domains, connected by a flexible linker (S2 Table).

After these calculations, the final designed sequences were altered by 15 to 39 mutations compared to the starting template (S1 Table). The designed proteins did not exhibit any full-length sequence homology with native human G-CSF, where sequence identity of the designs as aligned against G-CSF ranged between 7% and 15% of the overall template length. Furthermore, the topological features of the designs were much simpler as reflected in their relatively lower contact order and shorter chain lengths (S2 Table), where the diSohair designs particularly represent a radically simpler peptide topology that can support the binding epitope (Fig 1D).

### Biophysical and structural characterization

**Fig 2:**
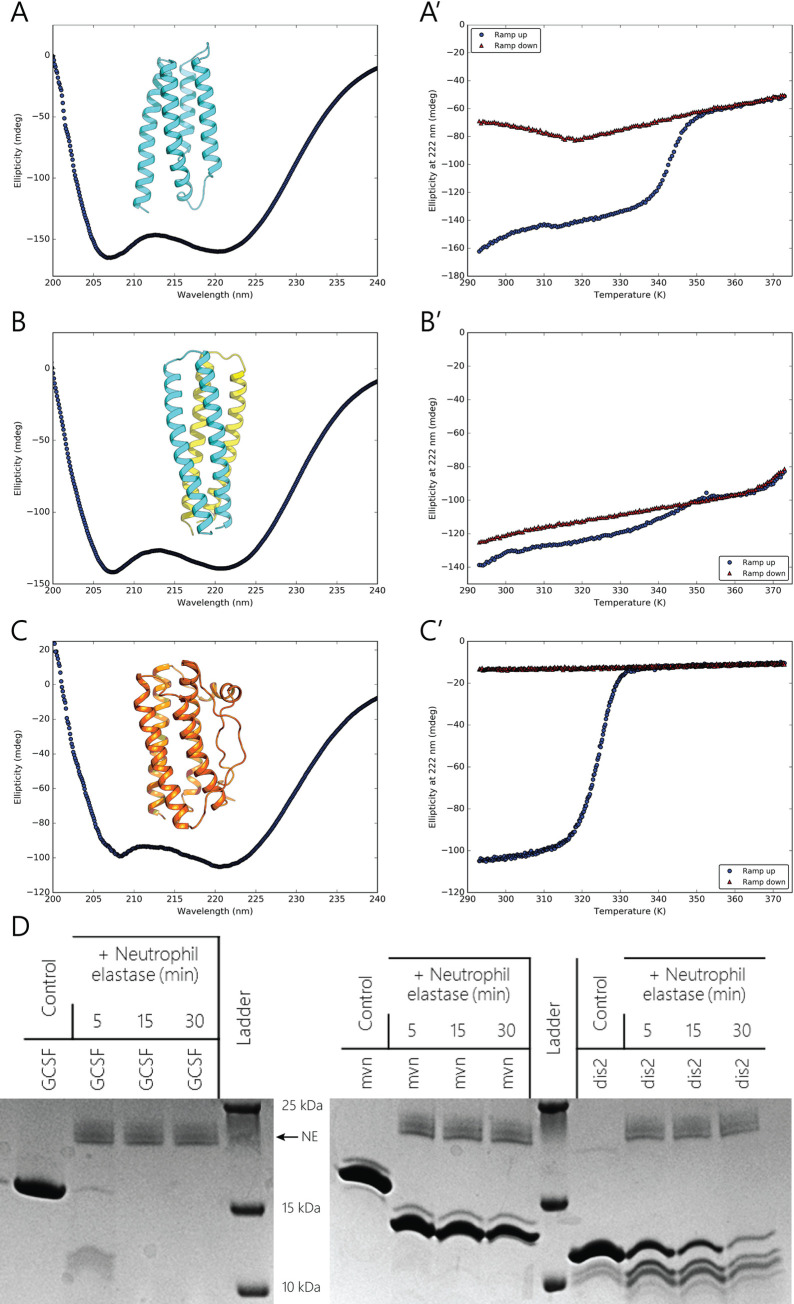
The designs are substantially more stable than rhG-CSF. Design models, CD spectra, and melting curves of the design models Moevan **(A, A’)**, diSohair2 **(B, B’)** as well as of rhG-CSF **(C, C’)** (S1 Data). Both design classes show strong helical ellipticity signals in comparison with rhG-CSF. Forward (blue) and reverse (red) melting curves show that Moevan has a melting temperature of 74°C and unfolds irreversibly (A’), whereas diSohair2 does not show any melting transition up to 100°C (B’). In contrast, G-CSF unfolds at 57°C and is irreversibly denatured (C’). Neutrophil elastase treatment of the designs diSohair2 (dis2) and Moevan (mvn) in comparison to rhG-CSF (GCSF). Representative images of Coomassie-stained gels are shown. CD, circular dichroism; G-CSF, granulocyte colony-stimulating factor; rhG-CSF, recombinant human G-CSF.

Starting with expression in *Escherichia coli*, all of the designs were soluble to yields above 15 mg/L culture. This is in contrast to rhG-CSF, which is insolubly expressed at a yield of 3.2 mg/L culture and has to be refolded. Circular dichroism (CD) spectra of Moevan and diSohair2 show alpha-helical content stronger than G-CSF at the same concentration (Fig 2). CD was also used to assess thermal unfolding of the most active designs, Moevan and diSohair2, and showed melting temperatures of 74°C and >100°C, respectively (Fig 2A and 2B). Moreover, diSohair2 reversibly regains its initial residual ellipticity upon cooling (Fig 2B). The melting curves of native rhG-CSF, however, showed irreversible thermal unfolding at a temperature of 57°C (Fig 2C).

To investigate whether enhanced thermostability can be of potential pharmacokinetic advantage, we tested the protease resistance of our designs in comparison to rhG-CSF. Neutrophil elastase (NE) is a serine protease that has broad substrate specificity. Secreted by neutrophils and macrophages during inflammation, NE enzymatically degrades G-CSF activity and constitutes a negative feedback loop against excessive cytokine-induced granulopoiesis [32,33]. Analyzing the products of NE treatment showed that rhG-CSF was almost entirely digested after a 5-minute treatment. Likewise, Moevan lost a large portion of its molecular weight upon NE treatment. diSohair2, however, was significantly more resistant to NE proteolytic activity, where most of the partial digestion occurred to terminal fragments, possibly representing the unstructured purification tag (Fig 2D).

To monitor the success of the design protocols, we determined the structures of 2 example proteins by NMR spectroscopy: Sohair and Moevan. These 2 proteins would represent the 2 folds investigated, as single-chain variants with minimal sequence symmetry. We applied the CoMAND method (Conformational Mapping by Analytical NOESY Decomposition), a protocol that we have recently developed to provide unbiased structure determination with minimal interpretation of the experimental data. In conventional NMR structure determination, spectra are analyzed to extract parameters—such as proton–proton distances and backbone torsion angles—that are used to generate structural models. This is usually done by converting these derived parameters into restraints for restrained MD simulations, i.e., models are driven to comply with the restraints by pseudo-energy terms in the simulations. In contrast, the CoMAND method selects models from a pool of unrestrained MD simulations to build an ensemble that best explains the input spectra. This is done via a quantitative R-factor expressing the deviation between experimental and back-calculated spectra [34]. In this way, any interpretation of spectra in terms of structural restraints is avoided. This in turn means that models are selected from simulations with inherently wider sampling of local conformational diversity without enforcing an average structure (Materials and methods).

**Fig 3:**
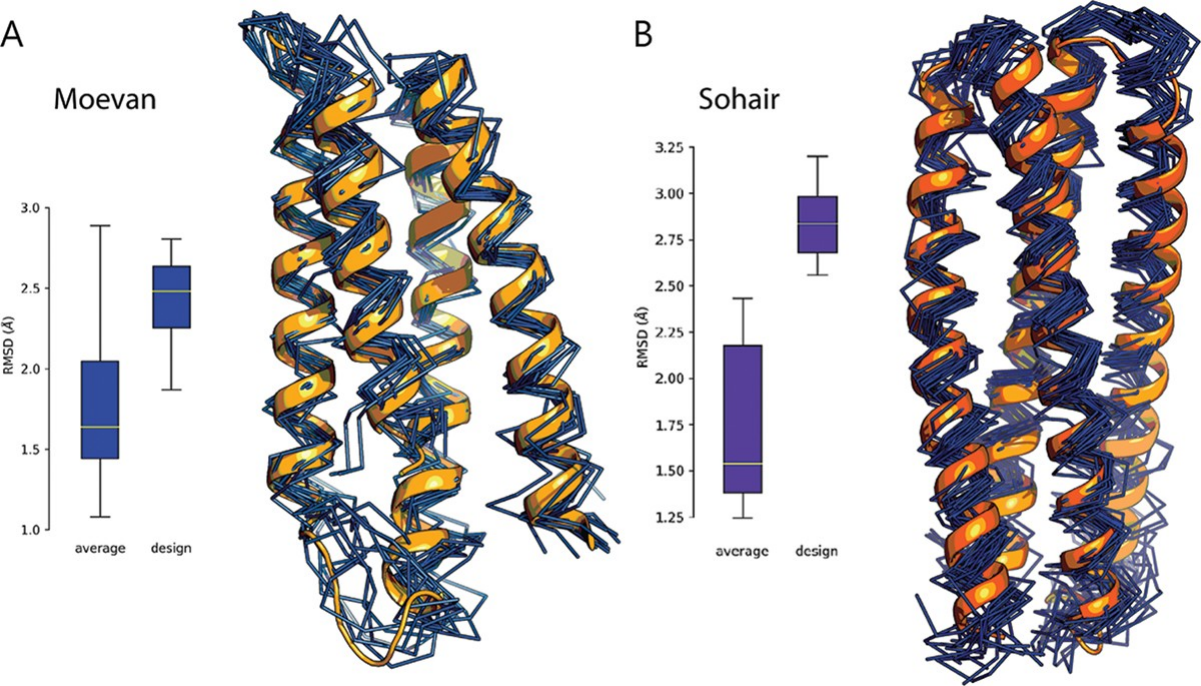
The design models show atomic-level agreement with their NMR structures. **(A)** Moevan showed an ensemble deviation from the average structure of 1.8 Å, and 2.46 Å from the design. The design model (orange) is shown against the NMR ensemble (dark blue) (PDB: 6Y06). **(B)** Sohair showed an ensemble deviation from the average structure of 1.78 Å and 2.85 Å from the design. The design model (orange) is shown against the NMR ensemble (dark blue) (PDB: 6Y07). Individual deviations are available in S1 Data.

For Moevan, The CNH-NOESY spectra provided sub-spectra for 102 amide protons, with those missing mainly due to unassigned resonances spanning 2 ranges (residues 1 to 8 and 65 to 67) where the latter stretch was a disordered loop in the template structure. We applied CoMAND factorization calculations to these sub-spectra, yielding backbone dihedrals both consistent with the values predicted from chemical shift profiles by TALOS-N [35] and having the lowest energy Rosetta *ab initio* folding decoy. Due to its high conformational heterogeneity, the refinement simulations for Moevan were carried out under a set of unambiguous distance restraints (S3 Table). During the frame-picking stage, R-factor minimization converged at 17 frames, 3 of which were rejected on the basis of distance restraint violations, leaving 14 frames constituting the final ensemble. The ensemble deviated by an average of 1.8 Å from the average structure and 2.5 Å from the design model (Fig 3A). Locally aligning the NMR ensemble to the G-CSF binding epitope stretches (residues 12 to 28 and 104 to 116) resulted in an RMSD of 1.0 Å.

For Sohair, we extracted 146 CNH-NOESY sub-spectra out of a total length of 154 residues (excluding the purification tag). Due to the significant pseudo-symmetry in the sequence and chemical environment, 29 of these had overlapped intensities. Performing CoMAND factorization on the nonoverlapped strips, we obtained backbone dihedrals consistent with TALOS-N predictions, which are in turn in line with the dihedral values of the lowest energy Rosetta *ab initio* folding decoy. The final, refined ensemble compiled by R-factor minimization yielded 19 frames (unambiguous restraints in S3 Table), with an RMSD of 1.8 Å from the average structure (S4 Table). Although the final ensemble has an average RMSD of 2.9 Å to the design model (Fig 3B), local alignment of the grafted epitope to G-CSF yields a considerably lower average RMSD of 1.5 Å.

Structural alignment of the solution structure of the grafted residues onto the native G-CSF epitope shows a good agreement with the positions and orientations of the side chains. The epitopes locally show lower ensemble and design deviation RMSD values compared to the global RMSD values. This indicates that these regions are more conformationally preserved, as both lie on a rigid helical segments close to the center of the bundles and away from the loops, which show larger deviations (S1 Fig).

### The designs possess granulocytic proliferative potential

**Fig 4:**
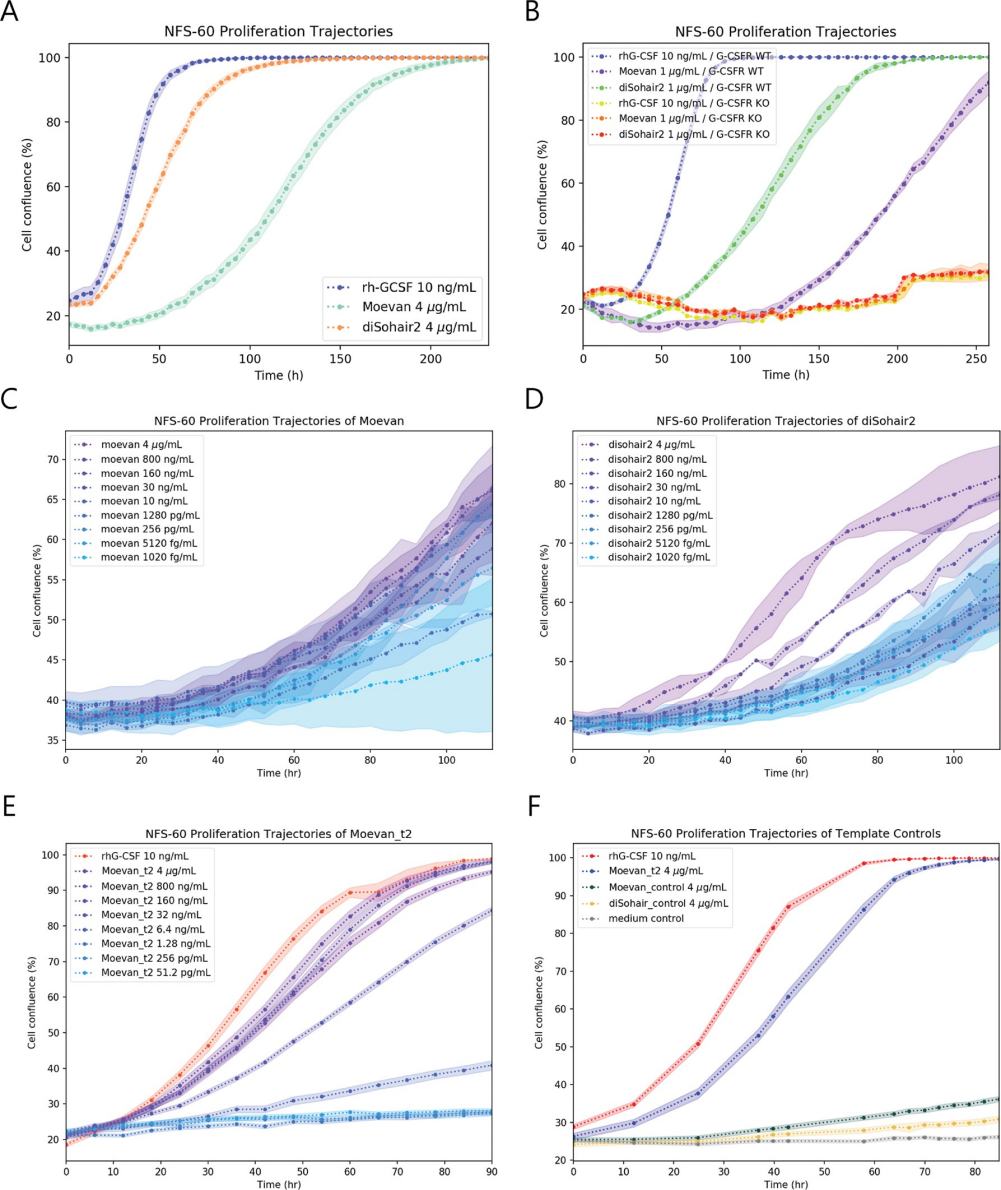
Proliferative activity of Moevan, Moevan_t2, and diSohair2 in murine NFS-60 cells. **(A)** Time-dependent proliferation trajectory of surface-immobilized NFS-60 cells over a 10-day treatment. Data points and shades represent mean and standard error (concentrations used: rhG-CSF: 532 pM; Moevan: 258 nM; diSohair2: 367 nM). **(B)** Surface-immobilized G-CSFR-deficient NFS-60 cells (G-CSFR KO), show abolished proliferative responses to either rhG-CSF or the designs. Data points and shades represent mean and standard error. **(C, D)** Dose- and time-dependent proliferation trajectories over a 5-day treatment of free-floating NFS-60 cells, under Moevan **(C)** or diSohair2 **(D)** treatments, respectively (concentration ranges: Moevan: 258 nM to 0.66 pM; diSohair2: 367 nM to 0.94 pM). Data points and shades represent the median and median absolute deviation from 3 separate measurements, respectively. **(E)** Dose- and time-dependent proliferation trajectories over 90 h for the tandemly repeated Moevan construct, Moevan_t2, compared against rhG-CSF. **(F)** The unmutated design templates (Moevan_control and diSohair_control) show no proliferative activity on NFS-60 cells. Data points and shades represent mean and standard error (S1 Data). G-CSFR KO, granulocyte colony-stimulating factor receptor knockout; G-CSFR WT, granulocyte colony-stimulating factor receptor wild type; rhG-CSF, recombinant human G-CSF.

In order to query the potential biological activity of the 4 experimentally tested designs, we used murine NFS-60 cells, a hematopoietic cell line that is routinely deployed to quantify granulopoietic proliferative potential [36]. Cell densities were assessed by a fluorescent redox-based assay 48 h after treatment with the designs or rhG-CSF, where the average *EC_50_* values (i.e., the concentration that gave half-maximal cell density response) ranged from micromolar to nanomolar (S2 Fig, S2 Table). While Sohair and diSohair1 showed the lowest activity among the 4 designs, the most active designs were Moevan and diSohair2 with *EC_50_* values of 16.05 nM ± 1.7 nM (249.3 ± 26.4 ng/mL) and 47.4 nM ± 2.95 nM (515.4 ± 32.1 ng/mL), respectively (S2 Fig). These values were substantially higher when compared to 1.7 pM ± 0.2 pM (40.0 ± 3.8 pg/mL) for rhG-CSF, which is in line with previous reports of filgrastim bioactivity [37]. We therefore set out to investigate the dose-response kinetics, in live imaging assays, which showed that these designs have significantly slower proliferation induction kinetics than rhG-CSF (Fig 4A and 4B and S1–S4 Movies) and that they continued to promote proliferation over a period of 5 days (Fig 4C and 4D). As we reasoned that site-II-mediated dimerization may be the mode of action of our designs, we set out to test a tandemly repeated version of Moevan; Moevan_t2 (S2 Table) might be more effective in receptor activation. The Moevan_t2 construct repeats the Moevan domain twice on a single protein chain using a flexible linker. End-point proliferation assays showed an *EC_50_* of 1.7 nM for Moevant_t2, which is approximately 10-fold higher activity than the monovalent Moevan design. This was also supported by dose- and time-dependent activity assays (Fig 4E and 4F, S2 Fig, and S2 Table).

As a control to test whether the proliferative activity was specifically endowed by the grafted binding epitope and not by the host protein, we generated and tested the unmutated original scaffold protein sequences of both diSohair2 (PDB: 5J73) and Moevan (PDB: 2QUP), herein referred to as Moevan_control and diSohair_control, respectively. Neither proteins showed any significant proliferative activity on NFS-60 cells (Fig 4F, S3 Fig, and S2 Table).

### Activation of G-CSFR signaling by the designs

To test whether the designs induce proliferation through G-CSFR signaling, we generated NFS60 G-CSFR knockout cells using CRISPR/Cas9-mediated gene editing. We synthesized a guide RNA (sgRNA) specifically targeting exon 4 of *CSF3R* (cut site: chr4 [+126,029,810: −126,029,810]) to introduce stop-codon or frameshift mutations in the extracellular part of all G-CSFR isoforms (S4A and S4B Fig) We generated pure G-CSFR knockout NFS-60 cell clones that carried 1 nucleotide deletion on each allele, as assessed by Sanger sequencing and tracking of indels by decomposition (TIDE) analysis. The resulting mutation caused a frameshift at the beginning of the ORF (S4C Fig). G-CSFR knockout NFS-60 cells did not respond to treatment with rhG-CSF, Moevan, or diSohair2 (Fig 4B), showing that the designs act via G-CSFR.

### The designs directly bind to human G-CSFR

**Fig 5:**
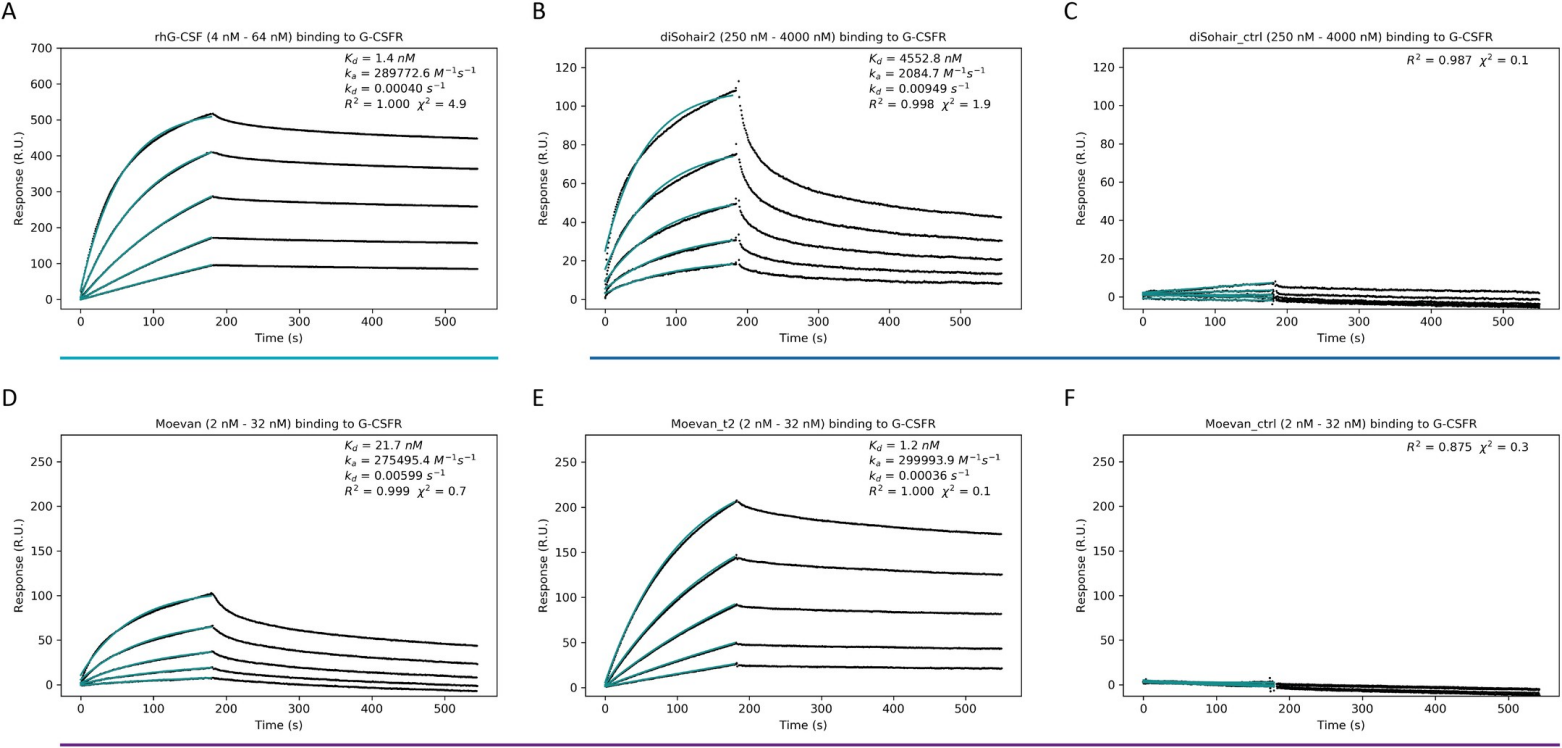
The designs directly bind the human G-CSF receptor. SPR sensograms of rhG-CSFR binding kinetics by **(A)** rhG-CSF, **(B)** diSohair2, **(C)** diSohair_control, **(D)** Moevan, **(E)** Moevan_t2, and **(F)** Moevan_control. Moevan_control and diSohair_control showed no measurable binding **(C, F)**. Cyan curves represent binding model fits, blue and purple lines indicate titrations conducted at the same concentration ranges for direct comparison (S1 Data). G-CSF, granulocyte colony-stimulating factor; rhG-CSF, recombinant human G-CSF; rhG-CSFR, recombinant human G-CSF receptor; SPR, surface plasmon resonance.

To characterize the kinetics and affinity of interactions between the designs and the G-CSF receptor, we performed surface plasmon resonance-based measurements for Moevan and diSohair2 in comparison to rhG-CSF (Fig 5, Table 1, and S5 and S6 Figs). Analysis of the kinetics across the injection dilution series, assuming 1:1 binding, resulted in dissociation constants (*K*_*d*_) of 4.5 μM, 21.0 nM, and 1.1 nM for diSohair2 (Fig 5B), Moevan (Fig 5D), and Moevan_t2 (Fig 5E), respectively. In comparison, the *K*_*d*_ determined for rhG-CSF was 1.1 nM (Fig 5A), in line with previous studies that have reported *K*_*d*_ values for the G-CSF:G-CSFR interaction between 200 pM using surface plasmon resonance (SPR) [38] and 1.4 nM using isothermal titration calorimetry (ITC) [39]. To test whether the grafted epitope residues mediate binding of the designs to the G-CSF receptor, we also performed SPR measurements for the Moevan and diSohair2 initial design templates (Moevan_control and diSohair_control, respectively), and no binding was observed (Fig 5C and 5F, S5C and S6C Figs). As bivalency influences the binding to and the activation of the G-CSFR, we also performed analytical size exclusion chromatography, which showed that diSohair2 assumes both dimeric and tetrameric forms, whereas Moevan is majorly monomeric with a minor dimeric fraction (S7 and S8 Figs).

**Table 1:** SPR binding parameters.

Analyte	*k*_*a*_ (*M*^−1^*s*^−1^)	*k*_*d*_ (*s*^−1^)	*K*_*d*_ (*M*)	*χ*^2^ (*R*.*U*.^2^)
rhG-CSF	(3.0 ± 0.3) × 10^5^	(4.9 ± 2.8) × 10^−4^	(1.1 ± 1.6) × 10^−9^	4.9
Moevan	(2.9 ± 0.4) × 10^5^	(5.9 ± 0.4) × 10^−3^	(2.1 ± 0.4) × 10^−8^	0.7
Moevan_t2	(3.1 ± 0.3) × 10^5^	(3.0 ± 3.2) × 10^−4^	(1.1 ± 1.1) × 10^−9^	0.1
diSohair2	(2.1 ± 0.1) × 10^3^	(9.5 ± 0.1) × 10^−3^	(4.5 ± 0.3) × 10^−6^	1.9

rhG-CSF, recombinant human G-CSF; SPR, surface plasmon resonance.

### The designs induce granulocytic differentiation of HSPCs in vitro

**Fig 6:**
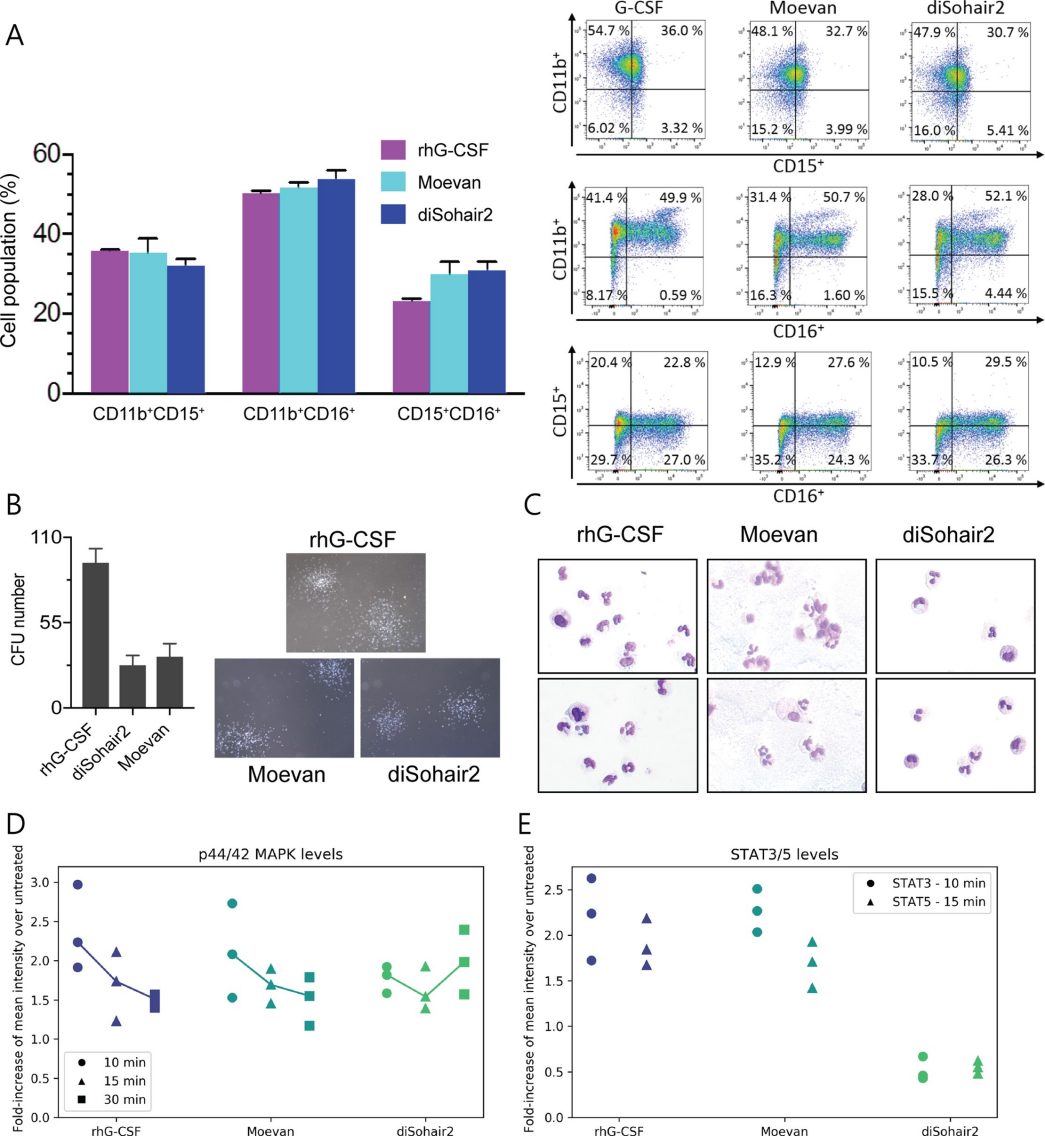
Evaluation of the biological activity of the designs in human donor-derived hematopoietic stem cells. **(A)** Representative FACS profiles (A) and neutrophil surface marker expression of treated CD34^+^ HSPCs as assessed after 14 d of culture. Data represent mean and standard deviation performed in triplicates from 2 different healthy donor samples. **(B)** Representative images of colonies induced by rhG-CSF, Moevan, and diSohair2 in CD34+ HSPCs after 10–14 days in culture and quantification of CFUs. Data represent mean and standard deviation of triplicates from 2 independent experiments. **(C)** Representative cytospin slide images of cells generated using liquid culture myeloid differentiation for 14 days. **(D, E)** Intracellular levels of phospho-ERK1/2 (p44/42 MAPK), phospho-STAT3, and phospho-STAT5 in CD34+ HSPCs treated with rhG-CSF or designs (see Materials and methods and S1 Data). CFU, colony-forming unit; HSPC, hematopoietic stem and progenitor cell; rhG-CSF, recombinant human G-CSF.

To evaluate the granulopoietic activity of the designs in human cells, we measured the granulocytic differentiation of CD34^+^ HSPCs from 2 healthy donors in the presence of 10 ng/mL of rhG-CSF, 10 μg/mL of diSohair2, or 10 μg/mL of Moevan (see Materials and methods). Fluorescence-activated cell sorting (FACS) analysis revealed differentiation of HSPCs into granulocytic cells (CD15^+^CD11b^+^, CD16^+^CD11b^+^, or CD15^+^CD16^+^ cells) in the presence of the designs to levels comparable to rhG-CSF (Fig 6A).

We further tested whether the designs induce the formation of myeloid colony-forming units (CFUs) from healthy donor HSPCs. Indeed, we observed myeloid CFUs in HSPC cultures in the presence of the designed proteins. Although the number of CFU colonies induced by Moevan and diSohair2 was much lower than the number stimulated by rhG-CSF, the typical myeloid cell morphology of CFUs was visible in all groups (Fig 6B).

The morphology of HSPCs-derived granulocytic cells was further evaluated in cytospin preparations on day 14 of culture. As expected, a majority of cells cultured in the presence of rhG-CSF or designs revealed the typical and highly specific morphology of neutrophilic granulocytes with multilobed nuclei (Fig 6C).

Binding of G-CSF to G-CSFR activates a rapid cascade of intracellular phosphorylation events, including downstream effectors such as STAT3, STAT5, or MAPK, which ultimately induces granulocytic differentiation of HSPCs. To test whether our designs directly induce G-CSFR signaling or whether they stimulate CFU formation through a secondary relay—e.g., by inducing cytokine production—we measured immediate phosphorylation targets of G-CSFR signaling in CD34+ HSPCs. Indeed, we found rapid tyrosine phosphorylation of p44/42 MAPK (Erk1/2) in HSPCs treated with Moevan or diSohair within minutes and to a similar degree as in rhG-CSF-treated cells (Fig 6D). At the same time, Moevan but not diSohair induced tyrosine phosphorylation of STAT3 and STAT5 proteins after 10 and 15 min of treatment, respectively (Fig 6E). We conclude that our designs directly induce intracellular G-CSFR downstream signaling.

## Discussion

In this work, we sought to design novel pharmacologically active proteins with optimized topologies and size and sequence composition, with the aim of achieving improved thermodynamic stability, kinetic stability, and solubility. As an example, here we chose to construct novel hematopoietic agents that activate the G-CSF/G-CSFR pathway, which induces the proliferation and differentiation of hematopoietic stem and progenitor cells into mature granulocytes. G-CSF/G-CSFR binding appears to be in the form of a 2:2 complex with 2 putative binding sites on the G-CSF surface, site II and site III. G-CSF interacts with its receptor primarily through site II and possibly through site III [27,40]. Crystallographic data pointed to the possibility of a 2:2 ligand-receptor complex in 2 different configurations, where 1 crystal structure involves a G-CSFR immunoglobulin-like (Ig-like) domain contact with G-CSF through site III [40,41]. This contact has been further corroborated by the similarity between the G-CSF:G-CSFR complex and the viral IL-6:gp130 complex and mutational studies of G-CSFR [28], and the direct binding of G-CSF to the isolated Ig-like domain from G-CSFR [42]. Alanine scanning experiments, however, showed a weak contribution to G-CSF activity from site III [27]. Site III exists along a helical stretch that interrupts a long loop stretch, where these long loop regions have been shown to possess a relatively low order parameter and fast rotational correlation time indicative of flexible regions (*S*^*2*^ = 0.57, τ_c_ = 0.42 ns) [43]. Therefore, we decided to focus on only grafting site II of G-CSF onto alternate scaffolds, presenting single and dual copies of the epitope.

We disembodied the G-CSF binding site II epitope and successfully rescaffolded it onto 2 different geometrically accommodating protein templates. Our design methods showed atomic-level accuracy, where the design model epitopes matched the determined solution structures at 0.95 Å and 1.5 Å for the representative designs Moevan and Sohair, respectively. Using this approach, we have neo-functionalized a bacterial protein with unknown function [30] and a *de novo* designed coiled coil that did not possess any prior function [31]. The designed proteins, unlike native rhG-CSF, lack any posttranslational modification, are produced at much higher yields, and have improved thermostability and lower contact order. The latter is expected to provide the designs with improved kinetic stability, i.e., faster folding and lower chances of misfolding. The thermodynamic stability of the designs is expected to decrease the probability of local unfolding, a prerequisite for proteolytic digestion. We tested the protease sensitivity of the 2 most active designs, and one of them (diSohair2) was significantly more protease-resistant than rhG-CSF.

While our receptor binding measurements showed that rhG-CSF binds G-CSFR at an affinity of around 1 nM, its proliferative activity in G-CSF-dependent cells was close to 2 pM. On the other hand, the binding affinities of the designs to G-CSFR were around 1 nM, 21 nM, and 5 μM for Moevan_t2, Moevan, and diSohair2, respectively. Despite the expectation that some of the binding epitope information may be lost as a result of the rescaffolding process (i.e., due to slightly altered molecular interaction fields), we argue that the efficiency of receptor dimerization is a major factor in determining the activity:affinity ratio. Receptor dimerization is more efficient when multiple binding epitopes exist on the same molecules and is also further enhanced when the epitopes are rigidly juxtaposed on the same protein domain in a productive orientation [44]. This can explain the improved Moevan_t2 activity (1.7 nM) compared to Moevan (18.6 nM) and the improved activity of diSohair2 (58 nM) compared to Sohair (280 nM). Particularly, the 2 proteins that rigidly present 2 binding epitopes on a single helical bundle are rhG-CSF (activity:affinity ratio: (0.002 nM)/(1.4 nM) = 1.4 × 10^−3^) and diSohair2 (activity:affinity ratio: (58 nM)/(4,552.8 nM) = 1.3 × 10^−2^), and for these 2 constructs, there is a much higher activity:affinity ratio than for Moevan_t2 (activity:affinity ratio: (1.7 nM)/(1.2 nM) = 1.4), where the repetition is achieved via a flexible linker.

Several studies have demonstrated that cytokine receptor activation not only depends on dimerization but also critically depends on the dimer orientation [45–47], which can strongly influence downstream signaling responses [48]. Based on our results, we speculate that a similar orientation sensitivity applies to G-CSFR activation. Whereas Moevan_t2 has a similar binding affinity as rhG-CSF, it still possesses lower activity, possibly due to the lack of a defined dimerization orientation owing to the flexible interdomain linker. Conversely, diSohair2 likely constitutes a very efficient receptor dimeriser, which could explain its high activity:affinity ratio compared to Moevan (although overall, it has lower receptor affinity and activity, most probably due to the higher RMSD deviation from the grafted epitope backbone and the smaller number of residues grafted onto its template compared to Moevan (11 and 14 residues, respectively; S1 Table)). This receptor-dimer orientation sensitivity might explain why Moevan and diSohair2 showed different phosphorylation kinetics of STAT3 and STAT5 (Fig 6E), in spite of the dually mounted site II epitope on diSohair2, which can more effectively create a 2:2 complex tethered through 2 site II epitopes.

In conclusion, the findings presented here demonstrate that exploiting the site II binding epitope can yield stable, soluble granulopoietic agents capable of proliferating and differentiating hematopoietic stem cells. The findings also point future research into investigating the impact of receptor dimerization efficiency and configuration on receptor activation magnitude, receptor internalization, downstream signaling responses, and differentiation efficacy. Prospectively, these orientation influences should be investigated through designing rigid assemblies presenting the binding epitopes in a variety of dimerizing (or oligomerizing) configurations.

### Accession numbers

The NMR structures of Moevan and Sohair were deposited in the PDB under the accession numbers 6Y06 and 6Y07, respectively. BMRB entries are deposited under accessions numbers 34488 and 34489, respectively.

## Materials and methods

### Protein design

The protein design calculations were done in 2 stages. The first stage was a geometric search, and the second was a design and optimization process. In the first stage, the PDB was systematically screened for accommodating structural scaffolds to host the essential site II residues, namely, K16, E19, Q20, R22, K23, D27, D109, and D112 (Fig 1A). The aim was to match backbone dihedrals and 3D backbone positions of the query residues to similar substructures in the PDB. To simplify the search space, the residues were assumed to lie on 2 discontinuous segments in the subject structures (i.e., segment 1: 16 to 27, segment 2: 109 to 112). A loop-grafting routine [29] was extended to generically search for pairs of structural segments disconnected by any number of intervening residues. The extended routine aims at finding minimizing arguments for 3 objective functions. The routines scan across a protein structure by defining the 4 sequence locations at the start and end of the 2 segments: Starting at sequence position *l*, the first segment ends at *l*+*k*_1_, a gap length *g* lets the start of the second segment position be at *l*+*k*_1_+*g*, and the window ends at the end of the second segment at *l*+*k*_1_+*g*+*k*_2_, where *k*_1_ and *k*_2_ are the sequence lengths of segments 1 and 2, respectively. The first function aims at finding the arguments of the minimum internal orientation differences between query and subject segment pairs as $\underset{l,g}{\mathrm{argmin}}\left|\left(s_{l}-s_{l+k_{1}})\times (s_{l+k_{1}+g}-s_{l+k_{1}+g+k_{2}})-(q_{1}-q_{1+k_{1}})\times (q_{2+k_{1}}-q_{2+k_{1}+k_{2}}\right)\right|$, where |∙| is the L^2^ norm, and ***s***_*l*_ or *q_1_* and *q_2_* is the position vector for the C_α_ at sequence position *l* for subject and query substructures, respectively. The second function aims at finding the arguments of the minimum of termini distance differences between query and subject segment pairs as $\underset{l,g}{\mathrm{argmin}}{\left(\left|q_{1+k_{1}}-q_{2+k_{1}}|-|s_{l+k_{1}}-s_{l+k_{1}+g}\right|\right)}^{2}+{\left(\left|q_{1}-q_{2+k_{1}+k_{2}}|-|s_{l}-s_{l+k_{1}+g+k_{2}}\right|\right)}^{2}$. The third function aims at finding the arguments of the minimum of the dihedral profiles difference across the corresponding amino acids between the query and subject substructures as $\underset{l,g}{\mathrm{argmin}}\frac{\sum \left(\phi_{q,i}⊖\phi_{s,i}\right)+\sum \left(\psi_{q,i}⊖\psi_{s,i}\right)}{2n}$, where ⊖ expresses the periodic angular deviation of the *ϕ* and *ψ* angles across corresponding amino acids indexed by *i* between the query and subject substructures. These 3 functions were applied in a tiered search scheme to systematically scan the PDB for candidate domains to host the disembodied residues. The top hits were re-ranked by their aligned RMSD to the query substructures and the smallest, topologically simplest, and posttranslational-modification-free hits were chosen for the design stage.

Sequence and conformer sampling was performed to the designs upon retrofitting the selected scaffolds with disembodied residues using the RosettaScripts framework [49]. In addition to an RMSD constraint on the binding epitope, a previously described core packing protocol was used [50]. This comprised steps of interleaved Monte Carlo sequence and side-chain and backbone conformer sampling iterations. The sequence sampling was directed to most core residues and to solvent-exposed hydrophobic residues. The scoring functions used were the *talaris2013* energy function [51] and the *packstat* packing score [52]. While the energy function was used to bias the sampling toward lower energy decoys, the top decoys were forwarded for further evaluation based on the packing quality, where the latter was further judged by the ruggedness of the radial distribution function *g*(*r*) as given by the definite integral $\int_{0}^{4}|\frac{dg(r)}{dr}|dr$. Novel loops were constructed through the automatic modeling [53] of 3- or 4-residue long loops covering all sequence combinations the residues G, D, P, S, L, N, T, E, and K. Molecular dynamics evaluation relied on repeated generalized-ensemble sampling (GS) [54] simulations, to estimate the conformational homogeneity of generated decoys across simulation replicas. All MD simulations relied on conjugate gradient minimization and a perturbation time step of 2 fs, under Langevin temperature control. A long-range interactions cutoff of 12.0 Å, a switching distance of 10.0 Å, and a Langevin temperature control set to 310 K with a damping coefficient of 1.0 ps^−1^ were used. Generalized Born solvation was used for GS simulations, neutralizing the system with 0.15 M sodium chloride. All simulations were either conducted using the OpenMM library [55] or the NAMD engine [56].

### Protein expression and purification

Synthetic genes encoding the designs were cloned into the pET28a(+) expression vector carrying a kanamycin resistance gene using *NdeI* and *XhoI* cloning sites in-frame with an N-terminal hexaHis-tag and a thrombin cleavage site (Synbio Technologies, Monmouth Junction, New Jersey, United States of America). The plasmids were used to transform chemically competent *E*. *coli* BL21(DE3) by heat shock. Transformed cells were grown in LB medium and induced with IPTG at OD600 of 0.5~1 with overnight expression at 25°C. For expression of labeled protein, a preculture in LB medium was grown, cells collected, washed twice in PBS buffer, and resuspended in M9 minimal medium (240 mM Na_2_HPO_4_, 110 mM KH_2_PO_4_, 43 mM NaCl), supplemented with 10 μM FeSO_4_, 0.4 μM H_3_BO_3_, 10 nM CuSO_4_, 10 nM ZnSO_4_, 80 nM MnCl_2_, 30 nM CaCl_2_, and 38 μM kanamycin sulfate, to an OD600 of 0.5~1. After 40 min of incubation at 25°C, 2.0 g ^15^N-labeled ammonium chloride (Sigma-Aldrich #299251) and 6.25 g ^13^C D-glucose (Cambridge Isotope Laboratories # CLM-1396) were added in a 2.5 L culture. After another 40 min, IPTG was added to a final concentration of 1 mM for overnight expression. Cells were collected by centrifugation at 5,000 *g* for 15 min, lysed by a Branson Sonifier S-250 (Fisher Scientific, Waltham, Massachusetts, USA) in hypotonic 50 mM Tris-HCl buffer supplemented with 1 tablet of the cOmplete protease cocktail (Sigma-Aldrich # 4693159001) and 3 mg of lyophilised DNase I (5,200 U/mg; Applichem # A3778). The insoluble fraction was pelleted by centrifugation at 25,000 *g* for 50 min, and the soluble fraction was passed through a 0.45 μm filter and directly applied to a Ni-NTA HisTrap column (GE Healthcare, Marlborough, Massachusetts, USA). For wild-type G-CSF, refolding extraction was performed from the insoluble fraction of the *E*. *coli* cell pellet by stirring in 8 M guanidinium chloride solution for 2 h at 4°C. The mixture was gradually diluted to 1 M guanidinium chloride in 4 steps over 4 h and loaded directly onto a Ni-NTA column. A 5 mL HisTrapFF immobilized nickel column (GE Healthcare Life Sciences #17-5255-01) was used for this purpose, washed consecutively by 30 mL 150 mM NaCl, 30 mM Tris buffer (pH 8.5) at 0, 30, and 60 mM imidazole. Fractions were collected by gradient elution at >60 mM imidazole. The eluate was concentrated using 3 kDa MWCO centrifugal filters (Merck Millipore # UFC901024) and loaded onto an equilibrated Superdex 75 gel filtration column (GE Healthcare Life Sciences #17517401). The gel filtration buffer used was 150 mM sodium phosphate buffer (pH 7.5) for NMR and CD transparency as well as cell culture compatibility. An Äkta Pure FPLC system (GE Healthcare Life Sciences) was used for all chromatography runs.

All in vitro experiments involving wild-type G-CSF were initially conducted using in-house purified G-CSF and later reproduced using USP filgrastim as a standard reference (Sigma-Aldrich #1270435–0.98MG).

### Thermostability analysis

CD spectra were recorded using a JASCO J-810 spectrometer. Samples (0.5 mL) with concentrations between 0.3 and 6 mg/mL of the respective proteins in 1× PBS buffer (pH 7.0) were loaded into 2 mm path length cuvettes. Spectral scans of mean residual ellipticity were measured at a resolution of 0.1 nm, across the range of 240 to 195 nm. The mean residual ellipticity at a wavelength of 222 nm across a temperature range of 20 to 100°C (with an increase of 1°C per minute) may be tracked in a melting curve.

### Protease sensitivity assays

Purified human neutrophil elastase was obtained from Enzo Life Science (#BML-SE284-0100). The elastase was reconstituted in PBS buffer (pH 7.0) to a stock concentration of 20 IU/mL. Digestion reactions were conducted in PBS buffer with final concentrations of 300 μg/mL of the tested protein and 1 U/mL of neutrophil elastase. The reaction mixture was incubated at 37°C and digestion samples were withdrawn, immediately mixed with SDS sample buffer (450 mM Tris HCl, 12% Glycerol, and 10% SDS) and flash-frozen in a liquid nitrogen bath to stop the reaction after 5, 15, and 30 min from the reaction start. Frozen samples were then heated at 85°C for 10 min before loading on Novex 16% Tricine Protein Gels (ThermoFisher Scientific # EC6695BOX). Protein gels were incubated overnight in fixing solution (30% ethanol, 10% acetic acid) and then stained using colloidal Coomassie dye (Serva; 35050).

### NMR structure determination

All spectra were recorded at 310 K on Bruker AVIII-600 and AVIII-800 spectrometers. Backbone sequential and aliphatic sidechain assignments were completed using standard triple resonance experiments, while aromatic assignments were made by linking aromatic spin systems to the respective C_β_H_2_ protons in a 2D-NOESY spectrum. Structures were calculated using the CoMAND method, which exploits the high accuracy that can be obtained in back-calculating NOESY spectra with indirect ^13^C dimensions [34]. The source of data for the protocol is a single 3D-CNH-NOESY spectrum, which is divided into 1D sub-spectra, each representing contacts to a single backbone amide proton. The protocol runs over several stages characterized by the nature and source of the pool of structures. In the first pass, the protein sequence is divided into a set of overlapping tripeptides that represent the local environment around the backbone amide proton in each 1D strip. The experimentally obtained 1D strips were decomposed against a library of spectra back-calculated by systematic sampling over a local dihedral angle space, yielding estimates of backbone and side chain dihedral angles for each residue. In the next stage, the information obtained on local dihedral angles is used to build full-length models—here using Rosetta *ab initio* folding—with successful models identified by low R-factors. In the last stage, the best scoring models are used to seed MD trajectories to produce a pool of thermodynamically relevant structures, and the final structure ensemble is selected from these models [57].

These sub-spectra are chosen from a search area centered on assigned ^15^N-HSQC positions and thus contain only cross-peaks to a specific amide proton. Residues with overlapping search areas were examined separately. In most cases, strips with acceptable separation of signals could be obtained. Where this was not possible, the residues were flagged as overlapped and a joint strip constructed by summing those at the estimated maxima of the respective components. Later stages of the protocol involve conformer selection aimed at minimizing a quantitative R-factor expressing the match between the experimental strips and back-calculated spectra or a fold-factor designed to isolate the contribution to the R-factor from long-range NOESY contacts [34].

For initial model building, unrestrained Rosetta *ab initio* [58] folding simulations were performed to generate 10,000 decoys. The corresponding CNH-NOESY spectra of these decoys were back-calculated to evaluate the structure-averaged fold-factors. The decoy with the lowest fold-factor was used to seed 5 independent unrestrained MD simulations. These refinement simulations were carried out using the CHARMM36 force field [59] in a Generalized Born implicit solvent. Trajectories of a total length of 2 μs were run, with frames collected every 100 ps. An initial refined ensemble was compiled through a global greedy minimization of the R-factor as previously described [34], which converged on a total of 17 frames for Moevan and 19 frames for Sohair. For Sohair, a set of unambiguous proton–proton NOEs were manually extracted from the CNH-NOESY and used as distance restraints. Three of these frames were rejected on the basis of restraint violations, leaving a final set of 14 models (S3 and S4 Tables).

### NFS-60 cell proliferation end point analysis

NFS-60 cells were cultured in IL-3-containing RPMI 1640 medium ready to use, supplemented with L-glutamine, 10% KMG-5, and 10% FBS (cls, cell line services). Before each assay, cells were pelleted and washed 3 times with cold non-supplemented RPMI 1640 medium. After the last washing step, cells were diluted at a density of 6 × 10^5^ cells/mL in RPMI 1640 containing glutamine and 10% FBS. In order to analyze cell proliferation, NFS-60 cells were grown in the presence of varying concentrations of G-CSF wild type and designed variants. For this, 5-fold dilution series were prepared from stock solutions of wild-type G-CSF (40 ng/mL) and the designs (40 μg/mL) in RPMI 1640 medium supplemented with glutamine and 10% FBS. A volume of 75 μL of each dilution were mixed with the same volume of washed cells in a 96-well plate yielding a final cell density of 3 × 10^5^ cells/mL and G-CSF concentrations varying from 0.00001 to 20 ng/mL for wild type and 0.01 to 20,000 ng/μL for the designs. Each 96-well plate contained triplicates of each dilution and the according blanks, including wells containing cells seeded in RPMI 1640 medium supplemented with L-glutamine, 10% KMG-5, and 10% FBS (cls, cell line services) and wells containing only medium. Following incubation for 48 h at 37°C and 5% CO_2_, 30 μL of the redox dye resazurin (CellTiter-Blue Cell Viability Assay, Promega, Madison, Wisconsin, USA) was added to the wells, and incubation was continued for another hour. Cell viability was measured by monitoring the fluorescence of each well at a H4 Synergy Plate Reader (BioTek, Winooski, Vermont, USA) using the following settings: excitation = 560 nm ± 9 nm, Emission = 590 nm ± 9 nm, read speed = normal, delay = 100 ms, measurements per data point = 10. The data were analyzed and curves were plotted applying a 4-parameter sigmoid fit using SigmaPlot (Systat Software, San Jose, California, USA). For calculation of percentage survival, the highest value of emission measured for each sample was set 100%.

### Evaluation of time- and dose-dependent effects on NFS-60 cell proliferation

NFS-60 cells were cultured in 96-well plates (2 × 10^4^ cells/well) in RPMI medium supplemented with 10% FCS, 2 mM L-Glutamine, 1 mM sodium pyruvate, 1% penicillin-streptomycin, different concentrations of rhG-CSF, Moevan, or diSohair2 in an IncuCyte S3 Live-Cell Analysis System (Essen Bioscience, Ann Arbor, Michigan, USA) at 37°C, 5% CO_2_ for 5 days. The analysis of cell proliferation was conducted using IncuCyte S3 Software.

### CRISPR/Cas9-sgRNA RNP-mediated CSF3R KO in NFS-60 cells

An sgRNA for knockout of the *CSF3R* gene (cut site: chr4 [+126.029.810: −126.029.810], NM_007782.3 and NM_001252651.1, exon 4, 112 bp after ATG; NP_031808.2 and NP_001239580.1 p.L38) was designed using CCTop at (http://crispr.cos.uni-heidelberg.de) [60]. Electroporation of NFS-60 cells was carried out using the Amaxa nucleofection system (SF cell line 4D-Nucleofector kit, #V4XC-2012) according to the manufacturer’s instructions. Briefly, 1 × 10^6^ cells were electroporated with assembled sgRNA (8 μg) and HiFi Cas9 nuclease protein (15 μg) (Integrated DNA Technologies, Coralville, Iowa, USA). Clonal isolation of single-cell–derived NFS-60 cells was performed by limiting dilution followed by an expansion period of 3 weeks. Genomic DNA of each single-cell–derived NFS-60 clones was isolated using QuickExtract DNA extraction solution (Lucigen #QE09050). PCR was carried out with mouse *CSF3R*-specific primers (forward: 5′-GGCATTCACACCATGGGGCACA-3′, reverse: 5′-GCCTGCGTGAAGCTCAGCTTGA-3′) and the GoTaq Hot Start Polymerase Kit (Promega, #M5006) using 2 μL of gDNA template for each PCR reaction. In vitro cleavage assay was done by adding 1 μM Cas9 RNP assembled by the same sgRNA used for the knockout experiment to 3 μL of each PCR product. The PCR reactions were incubated at 37°C for 60 min and run on a 1% agarose gel. The PCR products that showed no cleavage were purified by ExoSAP (ratio 3:1), which is a master mix of 1-part Exonuclease I 20 U/μL (Thermo Fisher Scientific, #EN0581) and 2 parts of FastAP thermosensitive alkaline phosphatase 1 U/μL (Thermo Fisher Scientific, #EF0651). Sanger sequencing of purified PCR products was performed by Microsynth and analyzed using the TIDE webtool [61].

### Surface plasmon resonance (SPR) binding assays

Multi-cycle kinetics experiments were performed on a Biacore X100 system (GE Healthcare Life Sciences). G-CSF Receptor (G-CSFR) (R&D systems 381-GR-050/CF) was diluted to 50 μg/mL in 10 mM acetate buffer (pH 5.0) and immobilized on the surface of a CM5 sensor chip (GE Healthcare 29149604) using standard amine coupling chemistry. The designs and rhG-CSF (USP RS Filgrastim, Sigma-Aldrich 1270435) were diluted in running buffer (10 mM HEPES pH 7.4, 150 mM NaCl, 3.4 mM EDTA, 0.005% v/v Tween-20). Analyses were conducted at 25°C at a flow rate of 30 μL/min. Five sequential 2-fold increasing concentrations of the sample solution (for diSohair2 and diSohair_control from 0.25 to 4 μM; for Moevan, Moevan_control, and Moevan_t2 from 2 to 32 nM; for rhG-CSF from 4 nM to 64 nM) were injected over the functionalized sensor chip surface for 180 s, followed by a 360 s dissociation with running buffer. At the end of each run, the sensor surface was regenerated with a 60 s injection of 10 mM glycine-HCl (pH 2.0). The reference responses and zero-concentration sensograms were subtracted from each dataset (double-referencing). Association rate (*k*_*a*_), dissociation rate (*k*_*d*_), and equilibrium dissociation (*K*_*d*_) constants were obtained using the linearization method described in [62]. Global fitting of the association curves to a 1:1 interaction model was performed using the following equation:

$$Г(t)={Г}_{GG}-{Г}_{GG}∙e^{-k_{obs}∙t}$$

Г(*t*) describes the surface load capacity over time (*t*), Г_*GG*_ is the equilibrium surface load capacity, and *k*_*obs*_ is the observed binding rate constant. The previous equation was rewritten as:

$$Г(t)=c+a∙e^{-b∙t},$$

where the parameters *a*, *b*, and *c* were fit to the data to minimize the value of *χ*^2^, which is evaluated by the expression:

$$\chi^{2}=\frac{\sum {\left({Г}_{fit}-{Г}_{obs}\right)}^{2}}{n-p},$$

where Г_*fit*_ is the Г(*t*) function with minimum sum of squared deviations from the observed sensogram Г_*obs*_, *n* is the number of data points (*n* = 900), and *p* is the number of parameters fitted by optimizer (*p* = 3). The optimization was performed using the Nelder–Mead method at a tolerance of 10^−12^ and a maximum number of 10^6^ iterations. The optimization bounds for parameters *a*, *b*, and *c* were (−7×10^2^, 0), (10^−4^, 10^−1^), (0, 7×10^2^), respectively. After fitting, the resulting *k*_*obs*_ values were plotted against the corresponding analyte concentrations (*C*) to perform a linear regression according to the following equation:

$$k_{obs}=k_{a}∙C+k_{d},$$

where *k*_*a*_ represents the slope, and *k*_*d*_ represents the y-axis intercept of the linear fit. The dissociation constant *K*_*d*_ was calculated as follows:

$$K_{D}=\frac{k_{d}}{k_{a}}$$

To measure the dispersion of a dataset, 5 additional linear fits of *k*_*obs*_(C) function were performed as described above, but excluding 1 analyte concentration at a time. Standard deviations of *k*_*a*_, *k*_*d*_, and *K*_*d*_ values were calculated as follows:

$$s=\sqrt{\frac{\sum {\left(x_{i}-\bar{x}\right)}^{2}}{N-1}},$$

where *x*_*i*_ is the value of *k*_*a*_, *k*_*d*_, or *K*_*d*_ derived from the i^th^ linear fit, $\bar{x}$ is the mean value of *k*_*a*_, *k*_*d*_, or *K*_*d*_, and *N* is the total number of performed linear fits (*N* = 5).

### In vitro granulocytic differentiation of HSPCs

Human CD34^+^ HSPCs were isolated from the bone marrow mononuclear cell fraction of 2 healthy donors by magnetic bead separation using the Human CD34 Progenitor Cell Isolation Kit (Miltenyi Biotech #130-046-703, Germany). CD34^+^ cells were cultured at a density of 2 × 10^5^ cells/mL in Stemline II Hematopoietic Stem Cell Expansion medium (Sigma Aldrich, #50192) supplemented with 10% FBS, 1% penicillin/streptomycin, 1% L-glutamine, and 20 ng/mL IL-3, 20 ng/mL IL-6, 20 ng/mL TPO, 50 ng/mL SCF, and 50 ng/mL FLT-3L. For the liquid culture granulocytic differentiation, expanded CD34^+^ cells (2 × 10^5^ cells/mL) were incubated for 7 days in RPMI 1640 GlutaMAX supplemented with 10% FBS, 1% penicillin/streptomycin, 5 ng/mL SCF, 5 ng/mL IL-3, 5 ng/mL GM-CSF, and 10 ng/mL of rhG-CSF, or 10 μg/mL of each design (Moevan or diSohair2). Medium was exchanged every second day. On day 7, medium was changed to RPMI 1640 GlutaMax supplemented with 10% FBS, 1% penicillin/streptomycin, and 10 ng/mL G-CSF, or 10 μg/mL of diSohair2 or Moevan. Medium was exchanged every second day until day 14. On day 14, cells were analyzed by flow cytometry using the following antibodies: mouse anti-human CD45 (Biolegend, #304036), mouse anti-human CD11b (BD, #557754), mouse anti-human CD15 (BD, #555402), and mouse anti-human CD16 (BD, #561248) on a FACSCanto II instrument.

Cell morphology was evaluated on cytospin preparations. On day 14 of culture, 10 × 10^4^ cells per cytospin slide were centrifuged at 400 *g* for 5 min at room temperature using a Thermo Scientific Cytospin 4 Cytocentrifuge. Wright-Giemsa-stained cytospin slides were prepared using Hema-Tek slide stainer (Ames, USA) and evaluated using a Nikon Inverted Microscope.

### Colony-forming units (CFUs) assay

CD34^+^ HSPCs at a concentration of 10,000 cells/mL medium were plated in 35 mm cell culture dishes in 1 mL Methocult H4230 medium (Stemcell Technologies, Vancouver, British Columbia, Canada) supplemented with 2% FBS, 10 μg/mL of 100× Antibiotic-Antimycotic Solution (Sigma) and 50 ng/mL of rhG-CSF or 1 μg/mL of diSohair2 or Moevan. Cells were cultured at 37°C, 5% CO_2_. Colonies were counted on day 10 to 14.

### Analysis of signaling effector activation in CD34^+^ HSPCs

CD34^+^ cells were cultured in Stemline II Hematopoietic Stemcell Expansion Medium (Sigma- Aldrich; #50192) supplemented with 10% FBS (Sigma-Aldrich; #F7524; batch-no. BCBW7154), 1% L-Glutamine (Biochrom; #K0283), 1% Pen/Strep (Biochrom; #A2213), and a premixed Cytokine Cocktail containing rh-IL3 (PeproTech; #200–03), rh-IL6 (Novus Biologicals; #NBP2-34901), rh-TPO, rh-SCF (both R&D Systems; TPO #288-TP200; SCF #255-SC-200), and rh-Flt-3L (BioLegend; #550606). The final concentration of IL-3, IL-6, and TPO was 20 ng/ml, and for SCF and Flt-3L 50 ng/ml. On day 6 of culture, serum- and cytokine-starved (3 h) CD34^+^ HSPCs were treated with the corresponding rhG-GCSF, Moevan, or diSohair2 for 10, 15, or 30 min, fixed in 4% PFA (Merck; #P6148) for 15 min at room temperature, and permeabilized for 30 min by slowly adding ice-cold methanol (C. Roth; #7342.1) to a final concentration of 90%. Cells were left overnight in methanol at −20°C and stained on the next day with specific antibodies recognizing phosphorylated signaling effectors (phospho-Stat3 (Tyr705) (D3A7) XP rabbit mAb (Cell Signaling; #9145); phospho-Stat5 (Tyr694) (C11C5) rabbit mAb (Cell Signaling; #9359), and phospho-p44/42 MAPK (Erk1/2) (Thr202/Tyr204) (E10) mouse mAb (Cell Signaling; #9106) or respective isotype control antibody (anti-mouse IgG (H+L), F(ab’)2 fragment (Alexa Fluor 488 Conjugate) (Cell Signaling; #4408; goat anti-rabbit IgG H+L (Alexa Fluor 488) (abcam; #ab150077) by incubation for 20 min on ice in PBS/2% BSA. After that, cells were washed twice in ice-cold PBS/2% BSA analyzed by FACS. To determine the background-corrected fluorescent signal from the corresponding phosphorylated proteins, the fluorescent signal of the appropriate isotype control estimated at each time point of stimulation was subtracted from the specific phospho-protein signal.

### Ethics statement

This study was approved by the ethics committee of the University Hospital Tuebingen (782/2019BO2), reported probes were analyzed anonymously.

## Supporting information

S1 Fig The grafted epitope solution structures match the G-CSF epitope. **(A)** Moevan solution structure (purple; PDB: 6Y06) shows the active site residues to align well to the native G-CSF epitope (green; PDB: 2D9Q). **(B)** Sohair solution structure (purple; PDB: 6Y07) shows the active site residues to align well to the native G-CSF epitope (green; PDB: 2D9Q). G-CSF, granulocyte colony-stimulating factor; PDB, Protein Data Bank.
(TIF)

S2 Fig Dose-response curves showing cell proliferation of NFS-60 cells in the presence of rhG-CSF (filgrastim), Moevan, diSohair2, or Moevan_t2. Cells were treated for 48 h and then subjected to a fluorescent redox-based cell viability assay (S1 Data). rhG-CSF, recombinant human G-CSF.
(TIF)

S3 Fig Dose-response curves showing cell proliferation of NFS-60 cells in the presence of Moevan_t2, Moevan_control, or diSohair_control. Cells were treated for 48 h and then subjected to a fluorescent redox-based cell viability assay (S1 Data).
(TIF)

S4 Fig Generation of the G-CSFR knockout NFS-60 cell line. **(A)** Schematic representation of the CRISPR/Cas9 design strategy to target and knock out 2 isoforms of G-CSFR (ENSMUSG00000028859). **(B)** Gene editing efficiency in the G-CSFR knockout NFS-60 cell line assessed by Sanger sequencing and sequence trace decomposition (TIDE). r^2^ is calculated to assess the goodness of fit by TIDE algorithm and r^2^ > 0.9 is considered as a reliable prediction. **(C)** Sequence verification of the G-CSFR knockout NFS-60 cell line confirms the disruption of the G-CSFR gene through a frameshift mutation at the beginning of the ORF. G-CSFR, granulocyte colony-stimulating factor receptor; TIDE, tracking of indels by decomposition.
(TIF)

S5 Fig **SPR sensorgrams of (A) rhG-CSF, (B) diSohair2, and (C) diSohair_control, binding to rhG-CSFR and their binding kinetics fit.** Sensograms and association phase fits are shown (left-side panes; data points: black dots, fits: cyan curves) against their respective *k*_*obs*_ fits (S1 Data). rhG-CSF, recombinant human G-CSF; rhG-CSFR, recombinant human G-CSF receptor; SPR, surface plasmon resonance.
(TIF)

S6 Fig **SPR sensorgrams of (A) Moevan, (B) Moevan_t2, and (C) Moevan_control, binding to rhG-CSFR and their binding kinetics fit.** Sensograms and association phase fits are shown (left-side panes; data points: black dots, fits: cyan curves) against their respective *k*_*obs*_ fits (S1 Data). rhG-CSF, recombinant human G-CSF; rhG-CSFR, recombinant human G-CSF receptor; SPR, surface plasmon resonance.
(TIF)

S7 Fig Analytical size-exclusion elution profile of Moevan (teal) shows a monomeric (major) and dimeric (minor) species. Calibration curve shown in gray.
(TIF)

S8 Fig Analytical size-exclusion elution profile of diSohair2 (teal) shows a dimeric and tetrameric species. Calibration curve shown in gray.
(TIF)

S1 Movie Representative time-lapse analysis of NFS-60 cells cultured without treatment (PBS only) for 10 d. (MP4)

S2 Movie Representative time-lapse analysis of NFS-60 cells cultured with Moevan treatment for 10 d. (MP4)

S3 Movie Representative time-lapse analysis of NFS-60 cells cultured with diSohair2 treatment for 10 d. (MP4)

S4 Movie Representative time-lapse analysis of NFS-60 cells cultured with rhG-CSF treatment for 10 d. (MP4)

S1 Table Sequence alignments of designs to their initial design templates, showing the migrated epitope residues highlighted in yellow. (DOCX)

S2 Table Protein sequences of the different designs and WT G-CSF. (DOCX)

S3 Table Unambiguous proton NOEs used to validate the final model. (DOCX)

S4 Table CoMAND ensemble structure statistics. (DOCX)

S1 Text Example routines used for design process sampling and scoring in this study. (DOCX)

S1 Data Numerical data displayed in the included figures. (XLSX)

## Abbreviations

CD: circular dichroism
CFU: colony-forming unit
CoMAND: Conformational Mapping by Analytical NOESY Decomposition
EPO: erythropoietin
G-CSF: granulocyte colony-stimulating factor
GS: generalized-ensemble sampling
HSPC: hematopoietic stem and progenitor cell
Ig: immunoglobulin
ITC: isothermal titration calorimetry
MD: molecular dynamics
NE: neutrophil elastase
PDB: Protein Data Bank
rhG-CSF: recombinant human G-CSF
RMSD: root-mean-square deviation
sgRNA: specific guide RNA
SPR: surface plasmon resonance
TIDE: tracking of indels by decomposition
