# Supplementary figures and images for: Design of novel granulopoietic proteins by topological rescaffolding
Source: PLoS Biol. 2020 Dec 22;18(12):e3000919. doi: 10.1371/journal.pbio.3000919 (PMC7755208; doi:10.1371/journal.pbio.3000919)

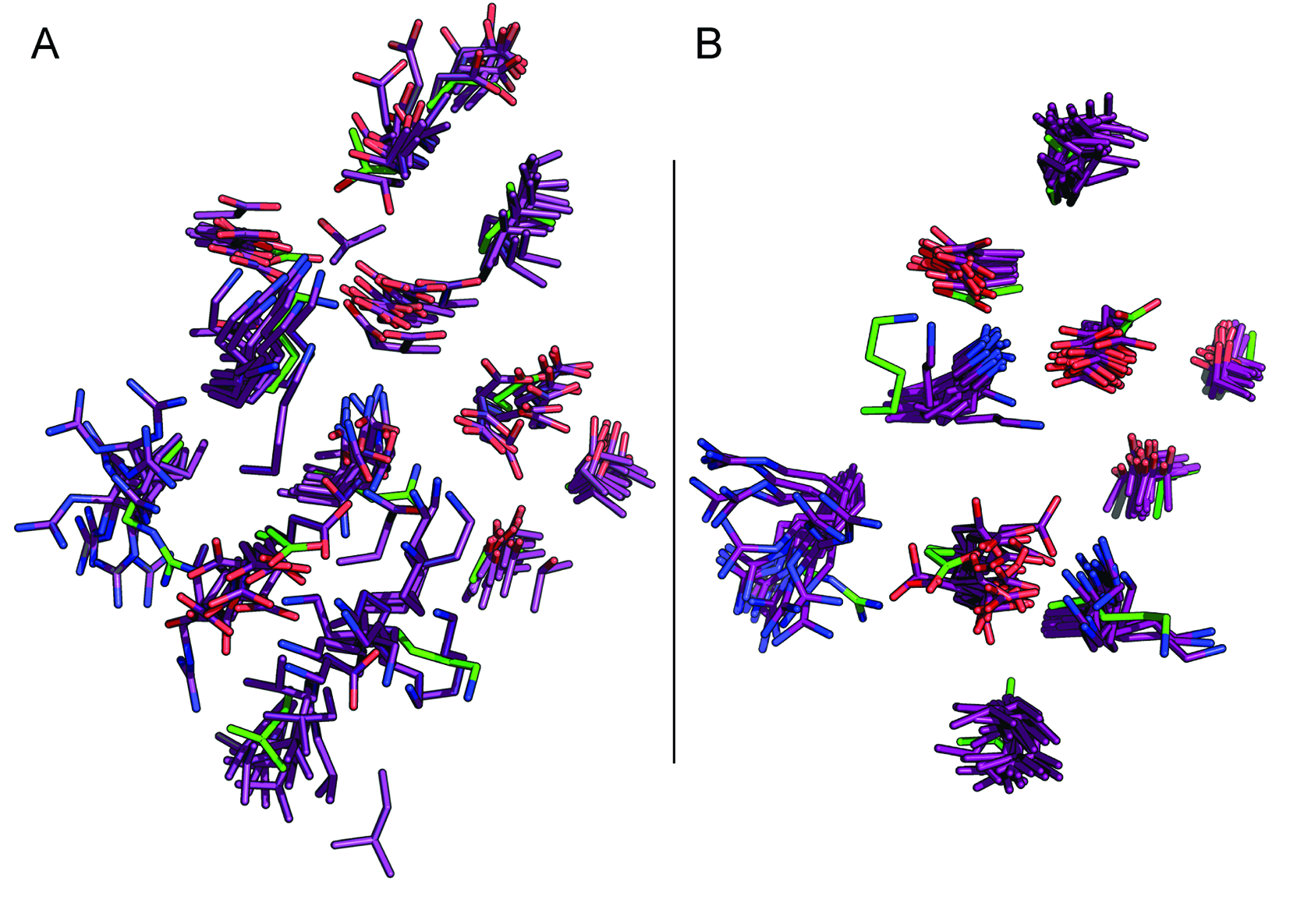

Supplement: S1 Fig — (A) Moevan solution structure (purple; PDB: 6Y06) shows the active site residues to align well to the native G-CSF epitope (green; PDB: 2D9Q). (B) Sohair solution structure (purple; PDB: 6Y07) shows the active site residues to align well to the native G-CSF epitope (green; PDB: 2D9Q). G-CSF, granulocyte colony-stimulating factor; PDB, Protein Data Bank. (TIF) [file pbio.3000919.s001.tif]

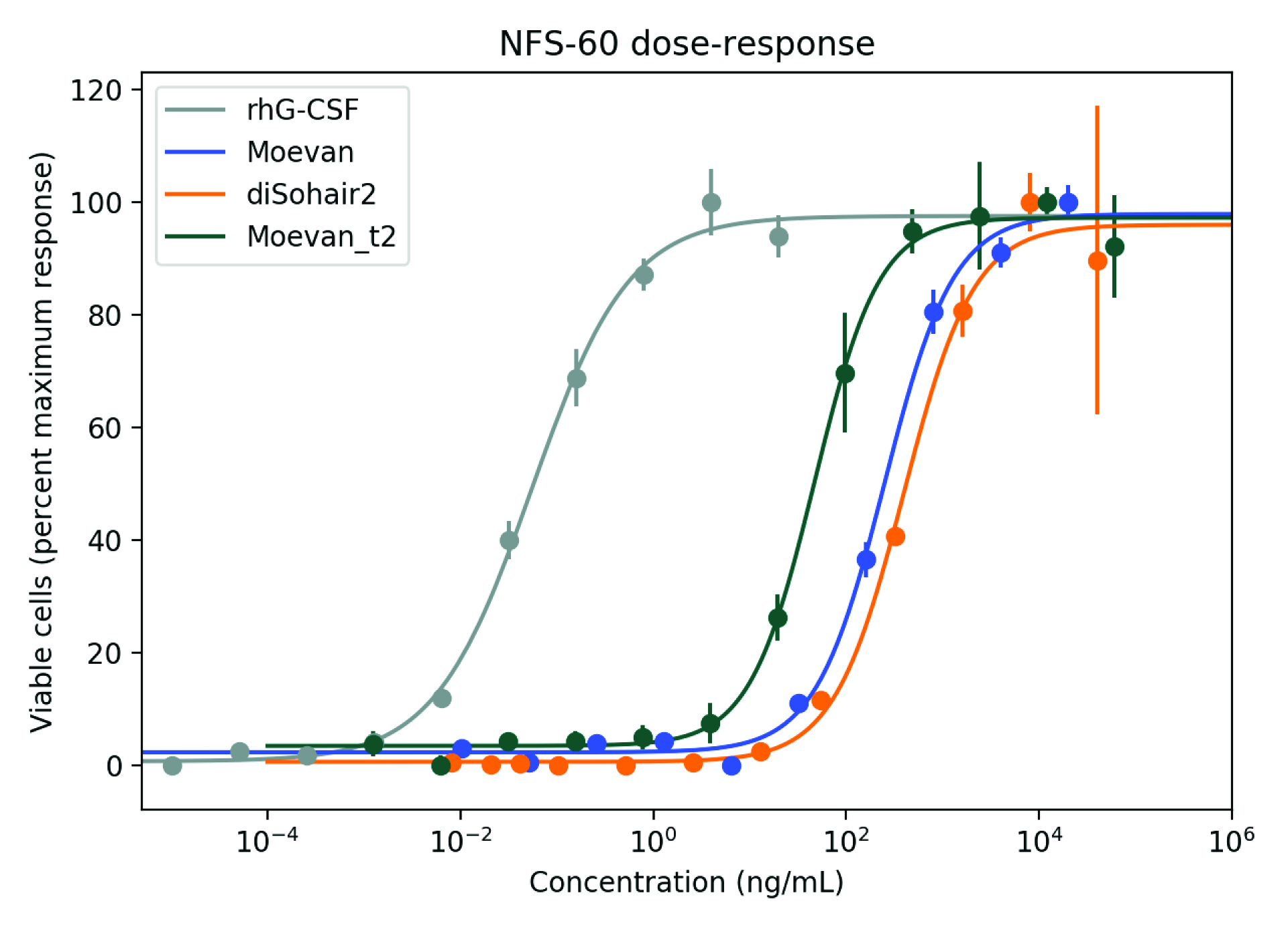

Supplement: S2 Fig — Cells were treated for 48 h and then subjected to a fluorescent redox-based cell viability assay (S1 Data). rhG-CSF, recombinant human G-CSF. (TIF) [file pbio.3000919.s002.tif]

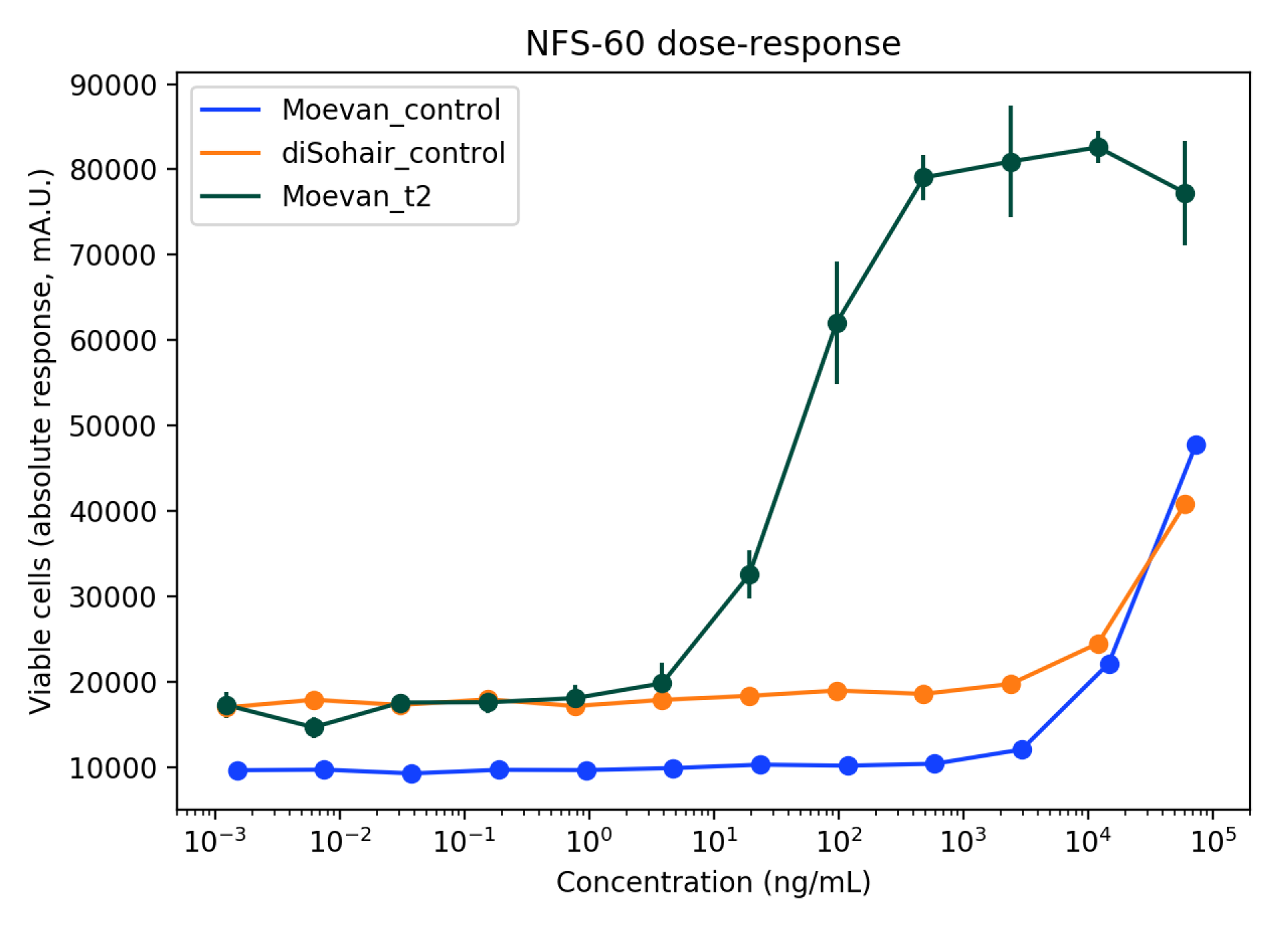

Supplement: S3 Fig — Cells were treated for 48 h and then subjected to a fluorescent redox-based cell viability assay (S1 Data). (TIF) [file pbio.3000919.s003.tif]

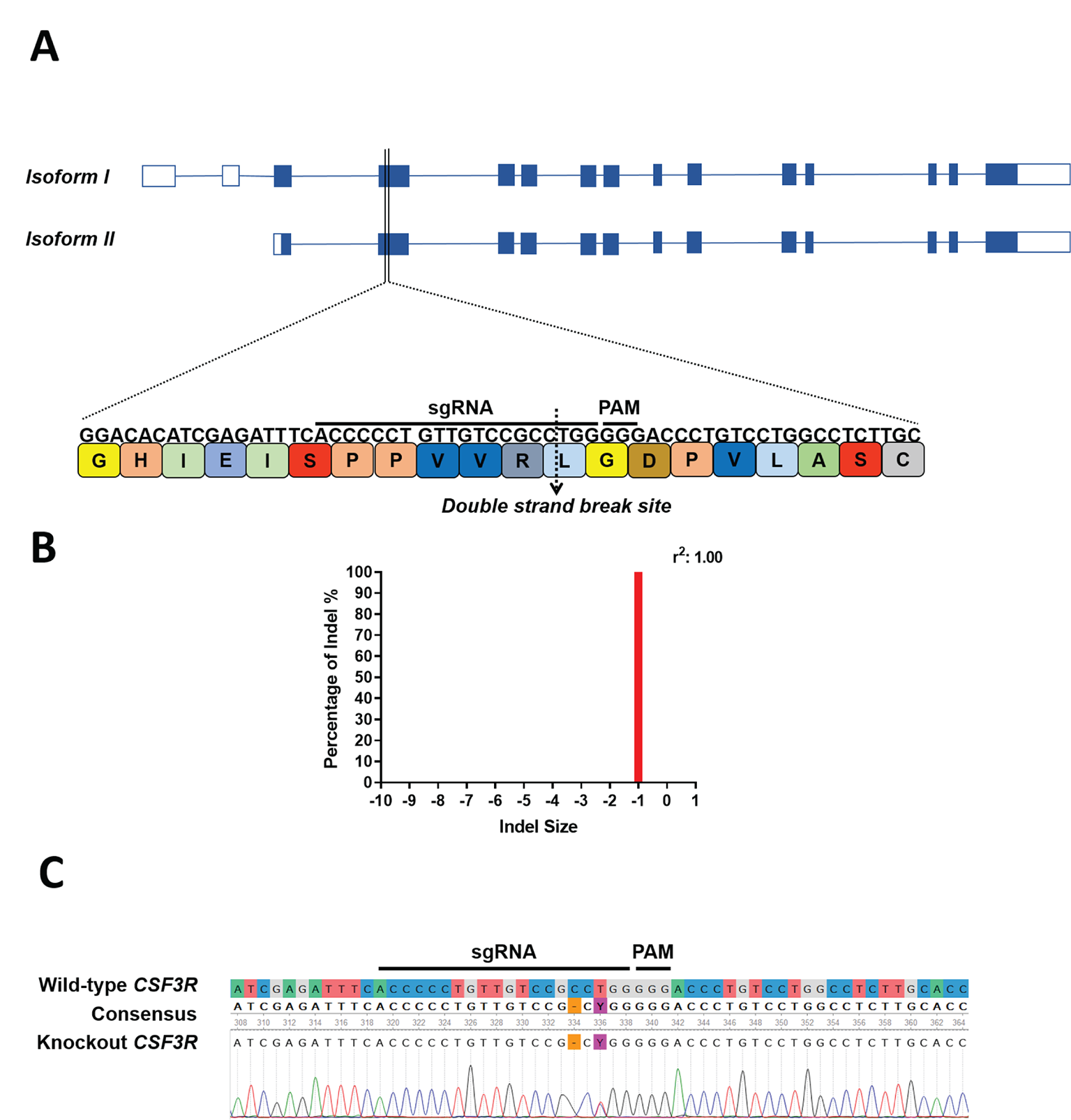

Supplement: S4 Fig — (A) Schematic representation of the CRISPR/Cas9 design strategy to target and knock out 2 isoforms of G-CSFR (ENSMUSG00000028859). (B) Gene editing efficiency in the G-CSFR knockout NFS-60 cell line assessed by Sanger sequencing and sequence trace decomposition (TIDE). r2 is calculated to assess the goodness of fit by TIDE algorithm and r2 > 0.9 is considered as a reliable prediction. (C) Sequence verification of the G-CSFR knockout NFS-60 cell line confirms the disruption of the G-CSFR gene through a frameshift mutation at the beginning of the ORF. G-CSFR, granulocyte colony-stimulating factor receptor; TIDE, tracking of indels by decomposition. (TIF) [file pbio.3000919.s004.tif]

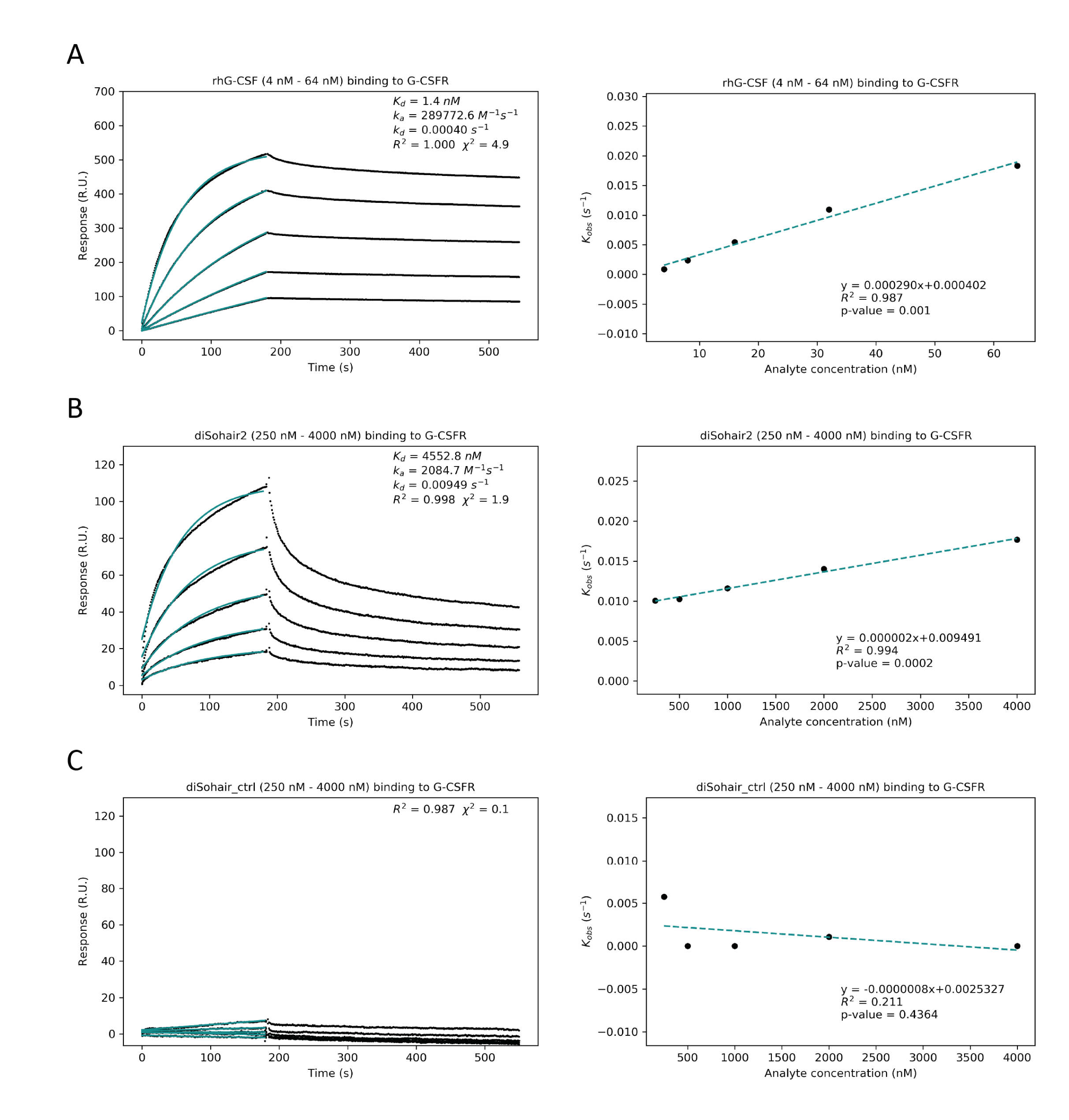

Supplement: S5 Fig — SPR sensorgrams of (A) rhG-CSF, (B) diSohair2, and (C) diSohair_control, binding to rhG-CSFR and their binding kinetics fit. Sensograms and association phase fits are shown (left-side panes; data points: black dots, fits: cyan curves) against their respective kobs fits (S1 Data). rhG-CSF, recombinant human G-CSF; rhG-CSFR, recombinant human G-CSF receptor; SPR, surface plasmon resonance. (TIF) [file pbio.3000919.s005.tif]

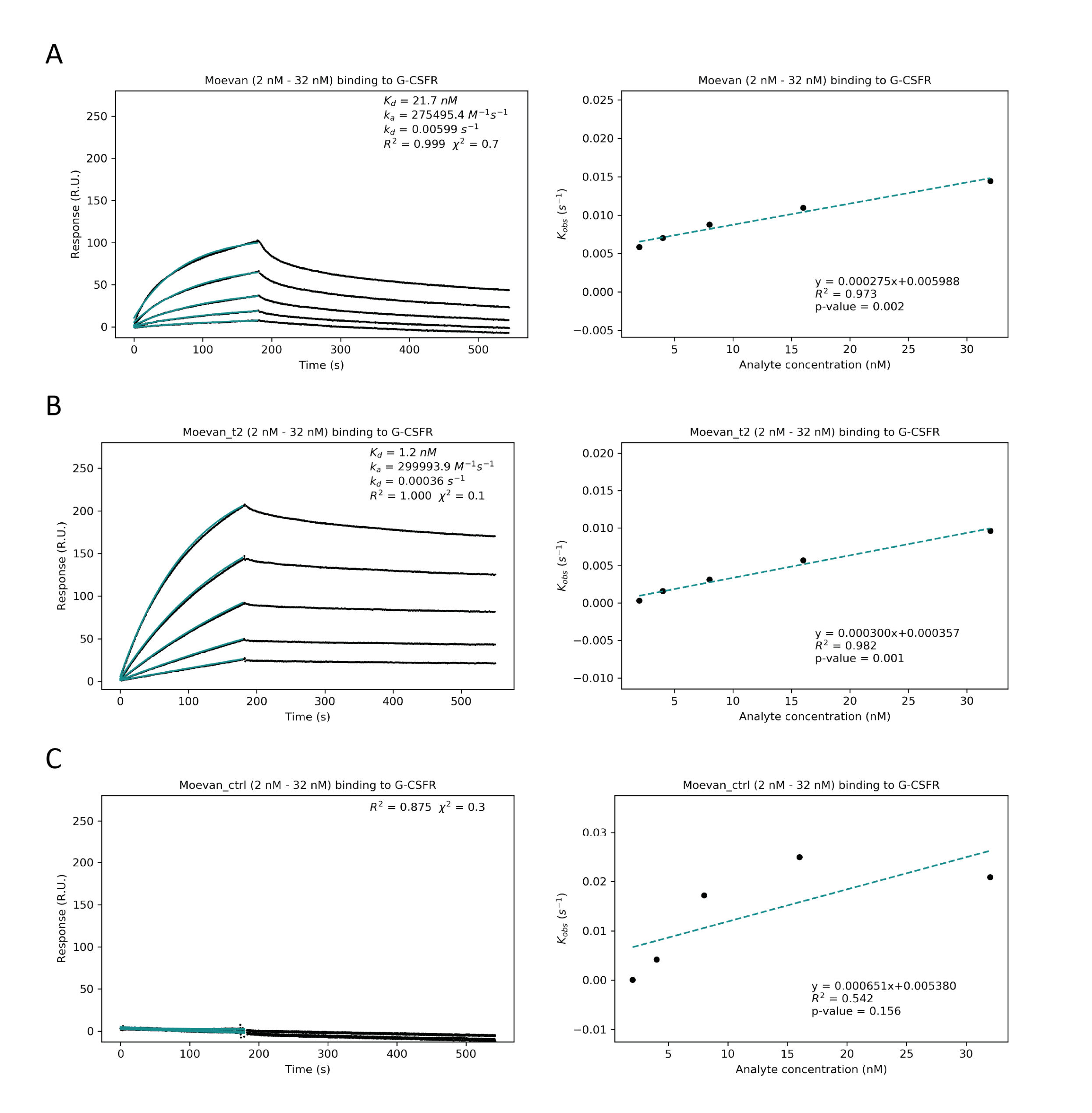

Supplement: S6 Fig — SPR sensorgrams of (A) Moevan, (B) Moevan_t2, and (C) Moevan_control, binding to rhG-CSFR and their binding kinetics fit. Sensograms and association phase fits are shown (left-side panes; data points: black dots, fits: cyan curves) against their respective kobs fits (S1 Data). rhG-CSF, recombinant human G-CSF; rhG-CSFR, recombinant human G-CSF receptor; SPR, surface plasmon resonance. (TIF) [file pbio.3000919.s006.tif]

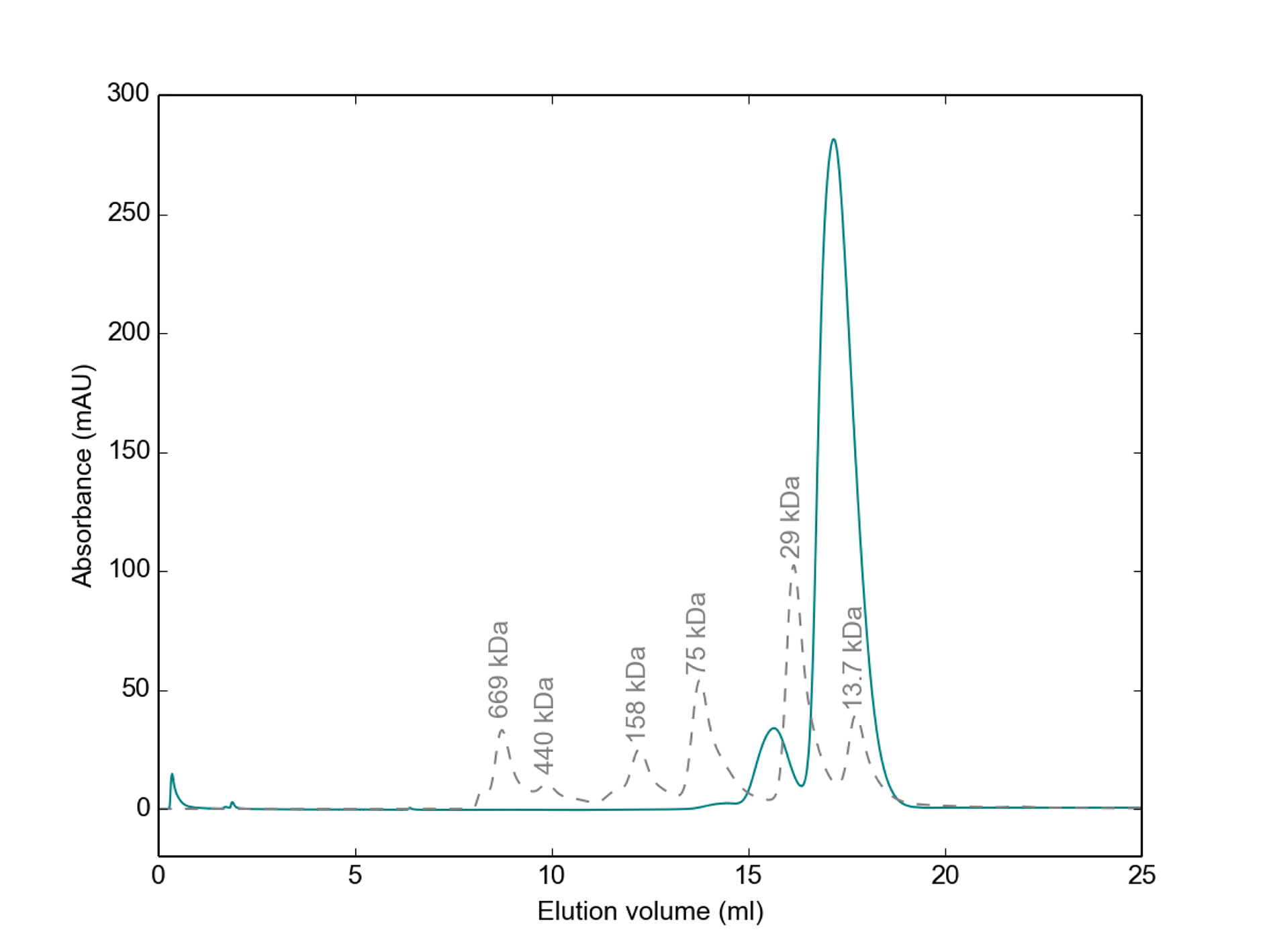

Supplement: S7 Fig — Calibration curve shown in gray. (TIF) [file pbio.3000919.s007.tif]

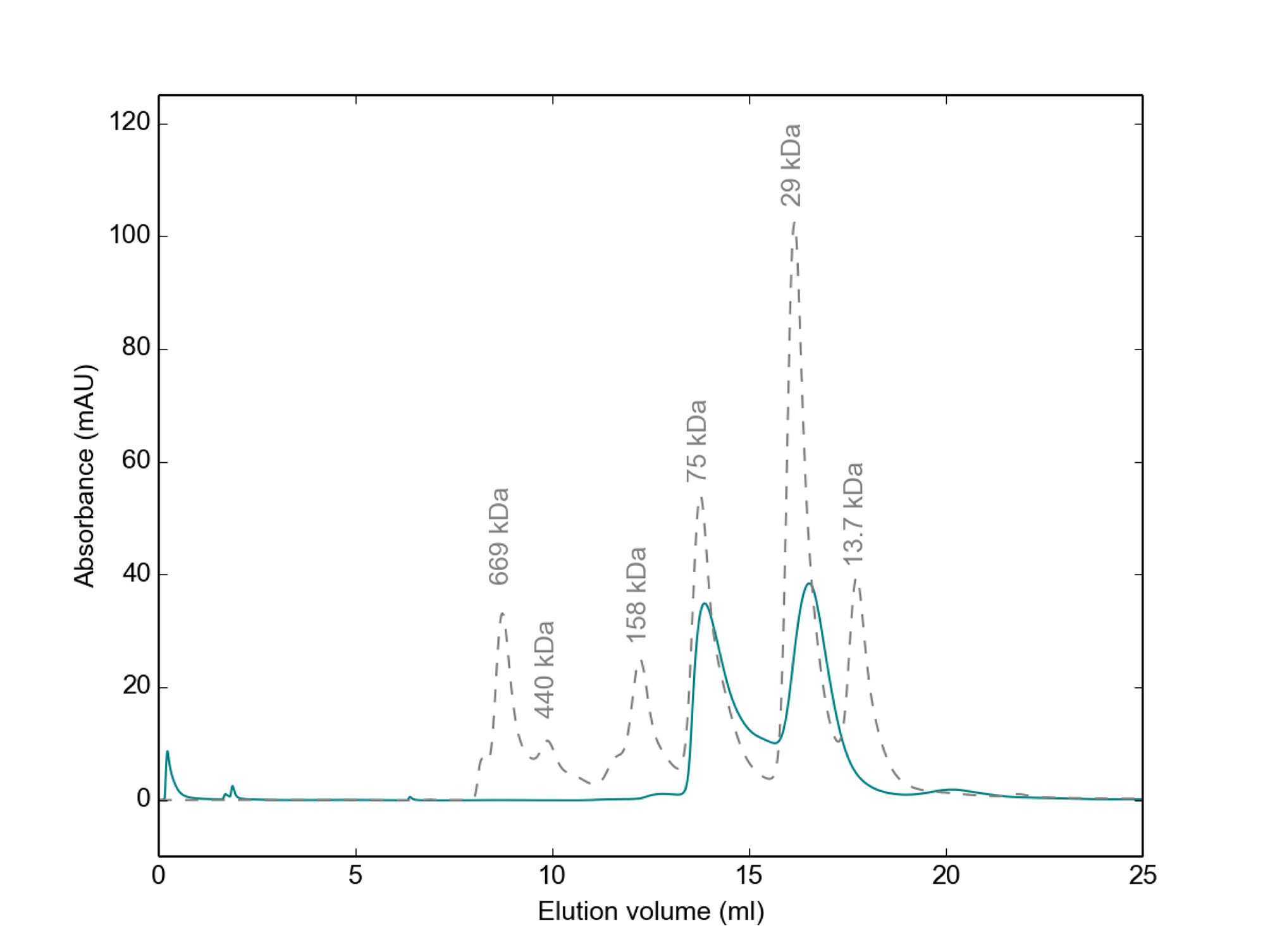

Supplement: S8 Fig — Calibration curve shown in gray. (TIF) [file pbio.3000919.s008.tif]
