# Supplementary material for: Design of novel granulopoietic proteins by topological rescaffolding
Source: PLoS Biol. 2020 Dec 22;18(12):e3000919. doi: 10.1371/journal.pbio.3000919 (PMC7755208; doi:10.1371/journal.pbio.3000919)
Supplement: S1 Table — (DOCX) [file pbio.3000919.s013.docx]

| **Table S1.** Sequence alignments of designs to their initial design templates, showing the migrated epitope residues highlighted in yellow. |
| --- |
| sohair MTSDYIIEQIQRKQEEARLKVEEMERKLEAVKEASKRGVSSDQLLNLILDLADIITTLIQ  5j73 MTSDYIIEQIQRDQEEARKKVEEAEERLERVKEASKRGVSSDQLLDLIRELAEIIEELIR  ************.***** **** *.:** ***************:** :**:** **:  sohair IIEESNEAIKELIKNQKGPTSDYIIEQIQRDQEEARKKVEEAEERLERVKEASKRGVSSD  5j73 IIRRSNEAIKELIKNQ-/-TSDYIIEQIQRDQEEARKKVEEAEERLERVKEASKRGVSSD  **..************ *****************************************  sohair QLLDLIRELAEIIEELIRIIRRSNEAIKELIKNQ  5j73 QLLDLIRELAEIIEELIRIIRRSNEAIKELIKNQ  ********************************** |
| diSohair1 MTSDYIIEQIQRKQEEARLKVEEMERKLEEVKEASKRGVSSDQLLNLILDLADIITTLIQ  5j73 MTSDYIIEQIQRDQEEARKKVEEAEERLERVKEASKRGVSSDQLLDLIRELAEIIEELIR  ************.***** **** *.:**.***************:** :**:** **:  diSohair1 IIEESNEAIKELIKNQ  5j73 IIRRSNEAIKELIKNQ  **..************ |
| diSohair2 MTSDYIIEQIQRKQEEARLKVEEQERKLEAVKEASKRGVSSDQLLNLILDLADIITTLIQ  5j73 MTSDYIIEQIQRDQEEARKKVEEAEERLERVKEASKRGVSSDQLLDLIRELAEIIEELIR  ************.***** **** *.:** ***************:** :**:** **:  diSohair2 IIEESNEAIKELIKNQ  5j73 IIRRSNEAIKELIKNQ  **..************ |
| moevan MEAAAAARDESAYLKLQEQMRKIDADAAALSETRTIEELDTFKLDVADFVTTVVQLAEEL  2qup SEVMGKQRDEKAYERLQALMSKIDDQGKLLSETRTIEELRKYKELVKEFVGDAVELGLRL  *. . ***.** :** * *** :. ********** .:* * :** .*:*. .*  moevan EHRFGRNRRGRTEIYKIVKEVDRKLLDLTDAVLAKEKKGEDILNMVAEIKALLINIYK  2qup EERRGFNRRGRTKIYKIVKEVDRKLLDLTDAVLAKEKKGLDILNMVGEIKGLLINIYA  *.* * ******:************************** ******.***.****** |
