# Supplementary material for: Design of novel granulopoietic proteins by topological rescaffolding
Source: PLoS Biol. 2020 Dec 22;18(12):e3000919. doi: 10.1371/journal.pbio.3000919 (PMC7755208; doi:10.1371/journal.pbio.3000919)
Supplement: S2 Table — (DOCX) [file pbio.3000919.s014.docx]

| **Table S2.** Protein sequences of the different designs and WT G-CSF | | |  |
| --- | --- | --- | --- |
| Name | Sequence | Absolute contact order | NFS-60  proliferation  average EC_50_ (ng/mL) |
| Moevan | MEAAAAARDESAYLKLQEQMRKIDADAAALSETRTIEELDTFKLDVADFVTTVVQLAEELEHRFGRNRRGRTEIYKIVKEVDRKLLDLTDAVLAKEKKGEDILNMVAEIKALLINIYK | 8.5 | 251 |
| diSohair1 | MTSDYIIEQIQRKQEEARLKVEEMERKLEEVKEASKRGVSSDQLLNLILDLADIITTLIQIIEESNEAIKELIKNQ | 4.5* | 2375 |
| diSohair2 | MTSDYIIEQIQRKQEEARLKVEEQERKLEAVKEASKRGVSSDQLLNLILDLADIITTLIQIIEESNEAIKELIKNQ | 4.5* | 519 |
| Sohair | MTSDYIIEQIQRKQEEARLKVEEMERKLEAVKEASKRGVSSDQLLNLILDLADIITTLIQIIEESNEAIKELIKNQKGPTSDYIIEQIQRDQEEARKKVEEAEERLERVKEASKRGVSSDQLLDLIRELAEIIEELIRIIRRSNEAIKELIKNQ | 9.4 | 5053 |
| Moevan_t2 | MEAAAAARDESAYLKLQEQMRKIDADAAALSETRTIEELDTFKLDVADFVTTVVQLAEELEHRFGRNRRGRTEIYKIVKEVDRKLLDLTDAVLAKEKKGEDILNMVAEIKALLINIYKGGGGSSGGGGSSGGGGSSGGGGSSEAAAAARDESAYLKLQEQMRKIDADAAALSETRTIEELDTFKLDVADFVTTVVQLAEELEHRFGRNRRGRTEIYKIVKEVDRKLLDLTDAVLAKEKKGEDILNMVAEIKALLINIYK | N/A | 47 |
| Moevan_control (PDB: 2QUP) | MEVMGKQRDEKAYERLQALMSKIDDQGKLLSETRTIEELRKYKELVKEFVGDAVELGLRLEERRGFNRRGRTKIYKIVKEVDRKLLDLTDAVLAKEKKGLDILNMVGEIKGLLINIYK | 8.5 | >50000 |
| diSohair_control (PDB: 5J73) | MTSDYIIEQIQRDQEEARKKVEEAEERLERVKEASKRGVSSDQLLDLIRELAEIIEELIRIIRRSNEAIKELIKNQ | 4.5* | >60000 |
| rhG-CSF | MSSLPQSFLLKCLEQVRKIQGDGAALQEKLCATYKLCHPEELVLLGHSLGIPWAPLSSCPSQALQLAGCLSQLHSGLFLYQGLLQALEGISPELGPTLDTLQLDVADFATTIWQQMEELGMAPALQPTQGAMPAFASAFQRRAGGVLVASHLQSFLEVSYRVLRHLAQP | 18.6** | 0.034 |
| * Absolute contact order calculated across a single chain.  ** Calculated for PDB entry 5GW9  (GGGGSS)_4_ flexible linker | | | |
|  | | |  |
