# Supplementary material for: Design of novel granulopoietic proteins by topological rescaffolding
Source: PLoS Biol. 2020 Dec 22;18(12):e3000919. doi: 10.1371/journal.pbio.3000919 (PMC7755208; doi:10.1371/journal.pbio.3000919)
Supplement: S3 Table — (DOCX) [file pbio.3000919.s015.docx]

| **Table S3.** Unambiguous proton NOEs used to validate the final model | | | | |
| --- | --- | --- | --- | --- |
| Residue number | Proton(s) | Residue number | Proton(s) | Distance (Å) |
| Moevan | | | | |
| 74 | HD1 | 53 | HG1+ | 4.8 |
| 74 | HE1 | 53 | HG1+ | 4.0 |
| 41 | HZ | 84 | HD2+ | 3.5 |
| 41 | HZ | 22 | HG2+ | 3.5 |
| 22 | HG2+ | 45 | HG2+ | 4.3 |
| 22 | HD1+ | 48 | HD2 | 4.3 |
| 91 | HG2+ | 29 | HD1+ | 4.8 |
| 48 | HD1 | 112 | HD2+ | 3.5 |
| 48 | HE1 | 112 | HB1 | 4.0 |
| 48 | HE1 | 112 | HD2+ | 4.0 |
| 48 | HD2 | 15 | HD2+ | 4.8 |
| 48 | HD2 | 18 | HB+ | 4.8 |
| 48 | HD2 | 18 | HG+ | 4.3 |
| 48 | HZ | 113 | HA | 4.5 |
| 112 | HD1+ | 48 | HD2 | 4.8 |
| 101 | HD1+ | 33 | HA | 4.8 |
| 101 | HG2+ | 30 | HA | 4.0 |
| 73 | HD1+ | 56 | HA | 4.8 |
| 108 | HG2+ | 41 | HE2 | 4.0 |
| 108 | HG2+ | 41 | HZ | 4.3 |
| 15 | HD1+ | 52 | HA | 4.0 |
| 15 | HD2+ | 52 | HA | 4.8 |
| Sohair | | | | |
| 102 | HN | 132 | HD+ | 4.8 |
| 99 | HN | 139 | HD+ | 4.8 |
| 130 | HN | 51 | HD1+ | 4.8 |
| 20 | HN | 140 | HD+ | 5.8 |
| 62 | HN | 136 | HD1+ | 4.8 |
| 68 | HN | 10 | HD+ | 5.8 |
