# Supplementary material for: Design of novel granulopoietic proteins by topological rescaffolding
Source: PLoS Biol. 2020 Dec 22;18(12):e3000919. doi: 10.1371/journal.pbio.3000919 (PMC7755208; doi:10.1371/journal.pbio.3000919)
Supplement: S4 Table — (DOCX) [file pbio.3000919.s016.docx]

**Table S4: CoMAND ensemble structure statistics**

| **R-Factors^1^** | Sohair | Moevan |
| --- | --- | --- |
|  |  |  |
| R_mean_ | 0.43 ± 0.11 | 0.40 ± 0.12 |
| Coverage^2^ | 146/154 | 102/117 |
| **Distance Restraints** |  |  |
| Number | - | 22 |
| RMSD (Å) | - | 0..002 |
| **Structure Quality** |  |  |
| Bonds (Å × 10^-3^) | 3.1 ± 0.2 | 2.2 ± 0.1 |
| Angles (°) | 0.72 ± 0.03 | 0.54 ± 0.02 |
| Impropers (°) | 1.43 ± 0.14 | 0.98 ± 0.06 |
| Ramachandran Map (%) | 98.3 / 1.6 / 0.1 | 98.4 / 1.6 / 0.0 |
| Sidechain Regularity (%) | 91.08 | 96.4 |
| Clash Score | 0 | 0 |
| Number of Structures | 19 | 14 |
| Ordered Residues | 3-153 | 4-64, 71-116 |
| Backbone Heavy Atom | 1.52 ± 0.36 | 1.58 ± 0.33 |
| All Heavy Atom | 1.99 ± 0.35 | 2.02 ± 0.31 |

^1^ R-factors averaged across the sequence (± SD) are given for the final ensemble compiled by global optimization (R_mean_).

^2^ The coverage refers to the number of residue used in factorization analysis, versus the total number expected from the sequence, excluding purification tags.

^3^ Determined by MOLPROBITY [[53](#_ENREF_53)]. The Ramachandran statistic lists the percentage of residues in favored / allowed / disfavored regions of the map (percentiles 98.0 / 99.8 / >99.8). Sidechain regularity lists the percentage in allowed sidechain rotamers (percentile 98.0). The clash score lists steric overlaps > 0.4 Å per 1000 atoms.

^4^The RMSD to the average structure based on superimposition over ordered residues, as defined in the table.
