# Supplementary material for: Design of novel granulopoietic proteins by topological rescaffolding
Source: PLoS Biol. 2020 Dec 22;18(12):e3000919. doi: 10.1371/journal.pbio.3000919 (PMC7755208; doi:10.1371/journal.pbio.3000919)
Supplement: S1 Text — (DOCX) [file pbio.3000919.s017.docx]

**Supplementary Text 1 | Example routines used for design process sampling and scoring in this study.**

Example design routine in rosettascripts:

<ROSETTASCRIPTS>

<SCOREFXNS>

<ramp_rep1 weights=talaris2013>

<Reweight scoretype=fa_rep weight=0.01/>

</ramp_rep1>

<ramp_rep2 weights=talaris2013>

<Reweight scoretype=fa_rep weight=0.1/>

</ramp_rep2>

<ramp_rep3 weights=talaris2013>

<Reweight scoretype=fa_rep weight=0.2/>

</ramp_rep3>

<ramp_rep4 weights=talaris2013>

<Reweight scoretype=fa_rep weight=1/>

</ramp_rep4>

</SCOREFXNS>

<FILTERS>

<EnergyPerResidue name=nrgy_per_res scorefxn=talaris2013 energy_cutoff=-1.3 whole_protein=1 confidence=0.8/>

<PackStat name=holes_1 threshold=%%pck_scr1%% chain=0 repeats=5/>

<PackStat name=holes_2 threshold=%%pck_scr2%% chain=0 repeats=5/>

</FILTERS>

<TASKOPERATIONS>

<RestrictToRepacking name=repackonly/>

<ReadResfile name=rrf filename=%%resfile%%/>

</TASKOPERATIONS>

<MOVERS>

<Backrub name=backrub/>

<PackRotamersMover name=des1 scorefxn=ramp_rep1 task_operations=rrf/>

<PackRotamersMover name=des2 scorefxn=ramp_rep2 task_operations=rrf/>

<PackRotamersMover name=des3 scorefxn=ramp_rep3 task_operations=rrf/>

<PackRotamersMover name=des4 scorefxn=ramp_rep4 task_operations=rrf/>

<FastRelax name=relax scorefxn=talaris2013 repeats=3/>

<ParsedProtocol name=design>

<Add mover_name=des1/>

<Add mover_name=des2/>

<Add mover_name=des3/>

<Add mover_name=des4/>

<Add mover=backrub/>

<Add mover_name=relax/>

<Add filter=holes_1/>

<Add mover_name=des4/>

</ParsedProtocol>

<GenericMonteCarlo name=mc_des_1 mover_name=design filter_name=nrgy_per_res scorefxn_name=talaris2013 trials=4/>

<GenericMonteCarlo name=mc_des_2 mover_name=design filter_name=holes_1 scorefxn_name=talaris2013 trials=1/>

</MOVERS>

<PROTOCOLS>

<Add mover_name=relax/>

<Add mover=backrub/>

<Add mover=backrub/>

<Add mover=mc_des_1/>

<Add mover=mc_des_2/>

<Add mover=backrub/>

<Add mover=backrub/>

<Add mover=backrub/>

<Add mover_name=relax/>

<Add mover=backrub/>

<Add filter=holes_2/>

<Add filter_name=nrgy_per_res/>

</PROTOCOLS>

</ROSETTASCRIPTS>

<!--

rosettascripts execution flags:

initial paramters: -ex1 -ex2 -parser:protocol cr_rds.xml -parser:script_vars resfile='mvn_resfile' pck_scr1='0.45' pck_scr2='0.5'

final paramters: -ex1 -ex2 -parser:protocol cr_rds.xml -parser:script_vars resfile='mvn_resfile' pck_scr1='0.55' pck_scr2='0.6'

-->

Example resfile for Moevan:

NATAA

start

15 A POLAR EX 1 EX 2 EX 3

18 A APOLAR EX 1 EX 2 EX 3

19 A POLAR EX 1 EX 2 EX 3

22 A POLAR EX 1 EX 2 EX 3

23 A POLAR EX 1 EX 2 EX 3

25 A POLAR EX 1 EX 2 EX 3

30 A ALLAAxc EX 1 EX 2 EX 3

31 A ALLAAxc EX 1 EX 2 EX 3

32 A ALLAAxc EX 1 EX 2 EX 3

33 A APOLAR EX 1 EX 2 EX 3

44 A POLAR EX 1 EX 2 EX 3

45 A POLAR EX 1 EX 2 EX 3

46 A APOLAR EX 1 EX 2 EX 3

48 A APOLAR EX 1 EX 2 EX 3

49 A POLAR EX 1 EX 2 EX 3

57 A PIKAA FLMI EX 1 EX 2 EX 3

65 A PIKAA AHNQ EX 1 EX 2 EX 3

69 A PIKAA FLIM EX 1 EX 2 EX 3

73 A PIKAA LIM EX 1 EX 2 EX 3

77 A PIKAA VLI EX 1 EX 2 EX 3

103 A PIKAA DE EX 1 EX 2 EX 3

108 A PIKAA VLIF EX 1 EX 2 EX 3

113 A POLAR EX 1 EX 2 EX 3

127 A POLAR EX 1 EX 2 EX 3

145 A ALLAAxc EX 1 EX 2 EX 3

Example of packing quality evaluation:

import sys

import numpy as np

from numpy import zeros, sqrt, where, pi, mean, arange, histogram

#####################################################################

class Atom():

def __init__(self, pdbatom_str):

self.idx = int(pdbatom_str[6:11])

self.name = pdbatom_str[12:16].strip()

self.resn = pdbatom_str[17:20].strip()

self.chain_id = pdbatom_str[21].strip()

self.resid = int(pdbatom_str[22:26])

x, y, z = float(pdbatom_str[30:38]), \

float(pdbatom_str[38:46]), float(pdbatom_str[46:54])

self.coords_arr = np.array([x, y, z])

#####################################################################

#####################################################################

def read_pdb(pdb_fn):

fh_in = open(pdb_fn, 'r')

atms_lst = [Atom(line) for line in fh_in.readlines() if line[:4] == "ATOM"]

fh_in.close()

return atms_lst

#####################################################################

#####################################################################

def gofr_3D_red(x, y, z, r_max, dr):

"""

input:

x array of x coordinates for all atoms

y array of y coordinates for all atoms

z array of z coordinates for all atoms

r_max outer diameter of largest spherical shell

dr resolution

output:

g(r) array containing the correlation function g(r)

radii array containing the radii of the

spherical shells used to compute g(r)

ref_idx indices of reference particles

"""

int_idx, = np.where(x != np.nan)

int_prtcls_num = len(int_idx)

edges = arange(0., r_max + 1.1 * dr, dr)

n_incr = len(edges) - 1

g = zeros([int_prtcls_num, n_incr])

radii = zeros(n_incr)

n_dnsty = 1.35

for p in range(int_prtcls_num):

index = int_idx[p]

d = sqrt((x[index] - x)**2 + (y[index] - y)**2 + (z[index] - z)**2)

d[index] = 2 * r_max

(result, bins) = histogram(d, bins=edges, normed=False)

g[p,:] = result / n_dnsty

g_avg = zeros(n_incr)

for i in range(n_incr):

radii[i] = (edges[i] + edges[i+1]) / 2.

r_out = edges[i + 1]

r_in = edges[i]

g_avg[i] = mean(g[:, i]) / (4.0 / 3.0 * pi * (r_out**3 - r_in**3))

return (g_avg, radii, int_idx)

#####################################################################

def main():

dr = 0.1

prtcl_rds = 1.5

r_max = 4

fn = sys.argv[1] # file name

try:

open(fn)

except:

print("failed to open designated file")

sys.exit

atms_lst = read_pdb(fn)

mol_xyz_arr = np.array([atm.coords_arr for atm in atms_lst])

g_r, r, ref_idx = gofr_3D_red(mol_xyz_arr[:,0], mol_xyz_arr[:,1], mol_xyz_arr[:,2], r_max, dr)

g_r = g_r/np.max(g_r)

dg_r = np.ediff1d(g_r)

adg_r = np.abs(dg_r)

iadg_r = np.sum(adg_r)

print(fn + ": " + str(iadg_r))

if __name__ == "__main__":

main()

Example NAMD tcl script for CHARMM scoring of decoys, where the potential energy in implicit solvent is averaged over the last 5000 time steps:

structure decoy.psf;

coordinates decoy.pdb;

set temperature 10;

set outputname decoy_out;

firsttimestep 0;

parameters charmm_36_prot.prm;

temperature $temperature;

gbis on;

alphaCutoff 12.0;

ionConcentration 0.15;

exclude scaled1-4;

cutoff 15.0;

switching on;

switchdist 12.0;

pairlistdist 18.0;

timestep 0.2 ;

rigidBonds all;

nonbondedFreq 1;

fullElectFrequency 1;

stepspercycle 1;

langevin on ;

langevinDamping 1 ;

langevinTemp $temperature;

langevinHydrogen off ;

outputName $outputname;

dcdfreq 10000;

outputEnergies 1;

minimize 5000;

reinitvels $temperature;

run 5000;

Example two-segment tertiary structure geometric matching routine:

Usage example with sample input and output is available at github at https://github.com/ElGamacy/two-seg_3ry_search

#!/usr/bin/python

import sys

import getopt

import numpy as np

def usage():

hlp_str = """Example two-segment geometric search routine

input:

-q query pdb file name <str>

-s subject pdb file name <str>

-d maximum spacing cutoff permitted between points 1->4 or 2->3 in Angstroms <float>

-a average (phi, psi) absolute dihedral deviations permitted in radians <float>

-1 segment 1 sequence length; segment between points 1->2 <int>

-2 segment 2 sequence length; segment between points 3->4 <int>

-b nth order of the first amino acid order in segment 1 to be considered <int>

-e reverse nth order of the last amino acid order in segment 2 to be considered <int>

-n minimum sequence gap length <int>

-x maximum sequence gab length <int>

example usage:

./geo_mtch_fex.py -q qry_fn.pdb -s sbj_fn.pdb -d 0.2 -a 0.2 -1 10 -2 10 -b 1 -e 1 -n 2 -x 50

"""

print(hlp_str)

def dihed(p1, p2, p3, p4, deg=False):

b_1 = p2 - p1

b_2 = p3 - p2

b_3 = p4 - p3

b_1_x_b_2 = np.cross(b_1, b_2)

b_2_x_b_3 = np.cross(b_2, b_3)

n_1 = b_1_x_b_2 / np.linalg.norm(b_1_x_b_2)

n_2 = b_2_x_b_3 / np.linalg.norm(b_2_x_b_3)

m_1 = np.cross(n_1, b_2/np.linalg.norm(b_2))

x = np.dot(n_1, n_2)

y = np.dot(m_1, n_2)

dhd = np.arctan2(y, x)

if deg: dhd = np.rad2deg(dhd)

return dhd

class atom():

def __init__(self, pdbatom_str):

self.idx = int(pdbatom_str[6:11])

self.name = pdbatom_str[12:16].strip()

self.resn = pdbatom_str[17:20].strip()

self.chain_id = pdbatom_str[21].strip()

self.resid = int(pdbatom_str[22:26])

x, y, z = float(pdbatom_str[30:38]), \

float(pdbatom_str[38:46]), \

float(pdbatom_str[46:54])

self.coords = np.array([x, y, z])

def read_pdb(fn):

fh_in = open(fn, 'r')

atms_lst = [atom(line) for line in fh_in.readlines() if "ATOM" \

in line]

return atms_lst

class res():

def __init__(self, res_atms_lst):

self.chain_id = res_atms_lst[0].chain_id

self.resid = res_atms_lst[0].resid

self.resn = res_atms_lst[0].resn

self.atms_lst = res_atms_lst

self.create_array()

def create_array(self):

tmp_coords_lst = [atm.coords for atm in self.atms_lst]

self.coords_arr = np.array(tmp_coords_lst)

self.atms_nms_lst = [atm.name for atm in self.atms_lst]

self.atms_nms_idx_dict = {atm.name : self.atms_lst.index(atm) \

for atm in self.atms_lst}

def gen_phi_psi(self, C_m1_coords, N_p1_coords):

self.phi = dihed(C_m1_coords, \

self.coords_arr[self.atms_nms_idx_dict["N"]], \

self.coords_arr[self.atms_nms_idx_dict["CA"]], \

self.coords_arr[self.atms_nms_idx_dict["C"]]) * -1

self.psi = dihed(self.coords_arr[self.atms_nms_idx_dict["N"]], \

self.coords_arr[self.atms_nms_idx_dict["CA"]], \

self.coords_arr[self.atms_nms_idx_dict["C"]], \

N_p1_coords) * -1

def update_coords(self):

for i in xrange(len(self.atms_lst)):

self.atms_lst[i].coords = self.coords_arr[i]

def get_del_sc(self):

tmp_bb_atms_lst = [atm for atm in self.atms_lst \

if (atm.name == "HN") or (atm.name == "N") \

or (atm.name == "CA") or (atm.name == "HA2") \

or (atm.name == "HA") or (atm.name == "C") \

or (atm.name == "O")]

return res(tmp_bb_atms_lst)

def bb_com(self):

tmp_res_bb = self.get_del_sc()

com = np.mean(tmp_res_bb.coords_arr, axis=0)

return com

def get_sc_atms(self):

tmp_sc_atms_lst = [atm for atm in self.atms_lst \

if (atm.name != "HN") and (atm.name != "N") \

and (atm.name != "CA") and (atm.name != "HA2") \

and (atm.name != "C") and (atm.name != "O")]

return tmp_sc_atms_lst

def subst_sc(self, inbnd_atms_lst):

tmp_mut_atms_lst = list()

tmp_bb_atms_lst = [atm for atm in self.atms_lst \

if (atm.name == "HN") or (atm.name == "N") \

or (atm.name == "CA") or (atm.name == "HA2") \

or (atm.name == "HA") or (atm.name == "C") \

or (atm.name == "O")]

tmp_mut_atms_lst.extend(tmp_bb_atms_lst)

return res(tmp_mut_atms_lst)

def reidx(self, idx_0=1):

c = idx_0

for i in xrange(len(self.atms_lst)):

self.atms_lst[i].idx = c

c += 1

def gen_resids(fn):

atms_lst = read_pdb(fn)

chid_lst = list(set([atm.chain_id for atm in atms_lst]))

resids_lst = list()

for chid in chid_lst:

resids_lst.append(list(set([atm.resid for atm in atms_lst \

if atm.chain_id == chid])))

res_lst = list()

for chn in resids_lst:

tmp_chn = list()

for resid in chn:

tmp_chn.append(res([atm for atm in atms_lst \

if atm.resid == resid]))

for i in xrange(len(tmp_chn)):

if i >= len(tmp_chn) - 2:

break

prev_res = tmp_chn[i]

cntr_res = tmp_chn[i+1]

next_res = tmp_chn[i+2]

tmp_chn[i+1].gen_phi_psi( \

tmp_chn[i].coords_arr[tmp_chn[i].atms_nms_idx_dict["C"]], \

tmp_chn[i+2].coords_arr[tmp_chn[i+2].atms_nms_idx_dict["N"]])

tmp_chn[0].phi=None

tmp_chn[0].psi=None

tmp_chn[-1].phi=None

tmp_chn[-1].psi=None

res_lst.append(tmp_chn)

return res_lst

def gapd_mtch_dual(frg_phi_psi, frg_com, sbj_phi_psi, sbj_com, \

up_seg, gap, dn_seg, tot_seg, cutoff_dhd_diff=0.32, \

cutoff_spcng=0.1, ornt_pos=[0, 2, -3, -1]):

hit_lst = list()

sbj_phi_psi = np.where(sbj_phi_psi == np.array(None), 0.0, sbj_phi_psi)

frg_phi_psi = np.where(frg_phi_psi == np.array(None), 0.0, frg_phi_psi)

frg_phi_psi = np.vstack((frg_phi_psi[ornt_pos[0]:ornt_pos[1]+1,:],frg_phi_psi[ornt_pos[2]-1:ornt_pos[3],:]))

frg_com = np.vstack((frg_com[ornt_pos[0]:ornt_pos[1]+1,:],frg_com[ornt_pos[2]-1:ornt_pos[3],:]))

frg_ends = np.linalg.norm(frg_com[ornt_pos[0],1:4]-frg_com[ornt_pos[-1],1:4])

frg_pre_ends = np.linalg.norm(frg_com[ornt_pos[1],1:4]-frg_com[ornt_pos[-2],1:4])

for i in range(sbj_phi_psi[0][0], sbj_phi_psi[-1][0] - tot_seg, 1):

tmp_sbjct = sbj_phi_psi[(sbj_phi_psi[:,0]>=i) & (sbj_phi_psi[:,0]<i+up_seg) | \

((sbj_phi_psi[:,0]>i+up_seg+gap) & (sbj_phi_psi[:,0]<=i+up_seg+gap+dn_seg))]

tmp_sbj_com = sbj_com[(sbj_com[:,0]>=i) & (sbj_com[:,0]<i+up_seg) | \

((sbj_com[:,0]>i+up_seg+gap) & (sbj_com[:,0]<=i+up_seg+gap+dn_seg))]

if len(tmp_sbjct) < len(frg_phi_psi): continue

# print(tmp_sbjct[:, 0])

res_pre_ends = np.linalg.norm(tmp_sbj_com[tmp_sbj_com[:,0]==tmp_sbjct[ornt_pos[1],0]][0][1:] - tmp_sbj_com[tmp_sbj_com[:,0]==tmp_sbjct[ornt_pos[-2],0]][0][1:])

spcng_1 = np.abs(res_pre_ends - frg_pre_ends)

if spcng_1 > cutoff_spcng:

continue

res_ends = np.linalg.norm(tmp_sbj_com[tmp_sbj_com[:,0]==tmp_sbjct[ornt_pos[0],0]][0][1:] - tmp_sbj_com[tmp_sbj_com[:,0]==tmp_sbjct[ornt_pos[-1],0]][0][1:])

spcng_2 = np.abs(res_ends - frg_ends)

if spcng_2 > cutoff_spcng:

continue

abs_diff = np.abs(tmp_sbjct[:,1:3] - frg_phi_psi[:,1:3])

abs_diff[abs_diff>=3.14] -= 3.14

avg_diff = np.sum(np.mean(abs_diff))

if np.sum(avg_diff) <= cutoff_dhd_diff:

hit_lst.append((np.sum(avg_diff), i))

else:

return None

hit_lst.sort()

return hit_lst

def main():

try:

opts, args = getopt.getopt(sys.argv[1:],"q:s:d:a:1:2:b:e:n:x:")

except getopt.GetoptError as e:

print(str(e))

usage()

sys.exit(2)

for o, a in opts:

if o == '-q':

qry_fn=str(a)

try:

open(qry_fn, "r")

except:

print("--USAGE ERROR\nUnable to open file\n%s" % qry_fn)

usage()

sys.exit(2)

if o == '-s':

sbj_fn=str(a)

try:

open(sbj_fn, "r")

except:

print("--USAGE ERROR\nUnable to open file\n%s" % sbj_fn)

usage()

sys.exit(2)

elif o == '-d':

cutoff_spcng=float(a)

if cutoff_spcng < 0.:

print("--USAGE ERROR\nNegative distance\n")

usage()

sys.exit(2)

elif o == '-a':

cutoff_dhd_diff=float(a)

if cutoff_dhd_diff < 0.:

print("--USAGE ERROR\nNegative absolute angular deviation\n")

usage()

sys.exit(2)

elif o == '-1':

seg_1_len=int(a)

if (seg_1_len < 1):

print("--USAGE ERROR\n--Invalid segment length")

usage()

sys.exit(2)

elif o == '-2':

seg_2_len=int(a)

if (seg_2_len < 1):

print("--USAGE ERROR\n--Invalid segment length")

usage()

sys.exit(2)

elif o == '-b':

seg_1_strt=int(a)

if (seg_1_strt < 1):

print("--USAGE ERROR\n--Invalid start index")

usage()

sys.exit(2)

elif o == '-e':

seg_2_rev_strt=int(a)

if (seg_2_len < 1):

print("--USAGE ERROR\n--Invalid segment length")

usage()

sys.exit(2)

elif o == '-n':

min_gap=int(a)

if (min_gap < 1):

print("--USAGE ERROR\n--Invalid minimum gap length")

usage()

sys.exit(2)

elif o == '-x':

max_gap=int(a)

if (max_gap < 1) or (max_gap < min_gap):

print("--USAGE ERROR\n--Invalid maximum gap length")

usage()

sys.exit(2)

try:

print("""--backbone geometric search with params:

query: %s

subject: %s

spacing cutoff: %f

dihedral deviation cutoff: %f

segment 1 length: %d

segment 2 length: %d

segment 1 start position: %d

segment end reverse position: %d

minimum sequence gap length: %d

maximum sequence gap length: %d

""" % (qry_fn, sbj_fn, cutoff_spcng, cutoff_dhd_diff, seg_1_len, \

seg_2_len, seg_1_strt, seg_2_rev_strt, min_gap, max_gap))

except Exception as e:

print(str(e))

print("--MISSING ARGUMENT(S) - REVISE USAGE")

usage()

sys.exit(2)

ornt_pos = [seg_1_strt-1, seg_1_strt+seg_1_len-2, \

-(seg_2_rev_strt+seg_2_len-1), -seg_2_rev_strt]

up_seg = seg_1_len

dn_seg = seg_2_len

frg_res_lst = gen_resids(qry_fn)[0]

frg_phi_psi = np.array([[r.resid, r.phi, r.psi] \

for r in frg_res_lst])

frg_com = np.array([np.hstack((r.resid, r.bb_com())) \

for r in frg_res_lst])

sbj_chns_lst = gen_resids(sbj_fn)

for sbj_res_lst in sbj_chns_lst:

sbj_phi_psi = np.array([[r.resid, r.phi, r.psi] \

for r in sbj_res_lst])

sbj_com = np.array([np.hstack((r.resid, r.bb_com())) \

for r in sbj_res_lst])

for gap in xrange(min_gap, max_gap):

# try:

tot_seg = up_seg + gap + dn_seg

h_lst = gapd_mtch_dual(frg_phi_psi, frg_com, \

sbj_phi_psi, sbj_com, up_seg, gap, dn_seg, tot_seg, \

cutoff_dhd_diff=cutoff_dhd_diff, \

cutoff_spcng=cutoff_spcng, ornt_pos=ornt_pos)

if h_lst: print("""subject chain length: %d, abs dihedral dev: %f, match start index %d, seg_1 length %d, gap length %d, seg_2 length %d""" % (len(sbj_phi_psi),h_lst[0][0],h_lst[0][1], seg_1_len, gap, seg_2_len))

if __name__ == "__main__":

main()
